# Superoxide Dismutase 1 Protects Hepatocytes from Type I Interferon-Driven Oxidative Damage

**DOI:** 10.1016/j.immuni.2015.10.013

**Published:** 2015-11-17

**Authors:** Anannya Bhattacharya, Ahmed N. Hegazy, Nikolaus Deigendesch, Lindsay Kosack, Jovana Cupovic, Richard K. Kandasamy, Andrea Hildebrandt, Doron Merkler, Anja A. Kühl, Bojan Vilagos, Christopher Schliehe, Isabel Panse, Kseniya Khamina, Hatoon Baazim, Isabelle Arnold, Lukas Flatz, Haifeng C. Xu, Philipp A. Lang, Alan Aderem, Akinori Takaoka, Giulio Superti-Furga, Jacques Colinge, Burkhard Ludewig, Max Löhning, Andreas Bergthaler

**Affiliations:** 1 CeMM Research Center for Molecular Medicine of the Austrian Academy of Sciences, Lazarettgasse 14 AKH BT25.3, 1090 Vienna, Austria; 2 Experimental Immunology, Department of Rheumatology and Clinical Immunology, Charité–Universitätsmedizin Berlin, Charitéplatz 1, 10117 Berlin, Germany; 3 German Rheumatism Research Center (DRFZ), a Leibniz Institute, 10117 Berlin, Germany; 4 Translational Gastroenterology Unit, Experimental Medicine Division Nuffield Department of Clinical Medicine, University of Oxford, John Radcliffe Hospital, OX3 9DU Oxford, UK; 5 Max Planck Institute for Infection Biology, Charitéplatz 1, 10117 Berlin, Germany; 6 Institute of Immunobiology, Cantonal Hospital St. Gallen, Rorschacherstrasse 95, 9007 St. Gallen, Switzerland; 7 Department of Pathology and Immunology, University of Geneva, Centre Médical Universitaire, 1 rue Michel Servet, 1211 Geneva, Switzerland; 8 Department of Neuropathology, University Medicine Göttingen, Robert-Koch Strasse 40, 37099 Goettingen, Germany; 9 Department of Medicine I for Gastroenterology, Infectious Disease and Rheumatology, Campus Benjamin Franklin, Charité-Universitätsmedizin Berlin, Hindenburgdamm 30, 12200 Berlin, Germany; 10 Department of Gastroenterology, Heinrich-Heine-University, Moorenstrasse 5, 40225 Düsseldorf, Germany; 11 Department of Molecular Medicine II, Heinrich Heine University, Universitätsstrasse 1, 40225 Düsseldorf, Germany; 12 Seattle Biomedical Research Institute, 307 Westlake Avenue North, Suite 500, Seattle, WA 98109-5219, USA; 13 Division of Signaling in Cancer and Immunology, Institute for Genetic Medicine, Hokkaido University, Sapporo, Hokkaido 060-0815, Japan; 14 Center for Physiology and Pharmacology, Medical University of Vienna, Lazarettgasse 14, 1090 Vienna, Austria

## Abstract

Tissue damage caused by viral hepatitis is a major cause of morbidity and mortality worldwide. Using a mouse model of viral hepatitis, we identified virus-induced early transcriptional changes in the redox pathways in the liver, including downregulation of superoxide dismutase 1 (*Sod1*). *Sod1*^*−/−*^ mice exhibited increased inflammation and aggravated liver damage upon viral infection, which was independent of T and NK cells and could be ameliorated by antioxidant treatment. Type I interferon (IFN-I) led to a downregulation of *Sod1* and caused oxidative liver damage in *Sod1*^*−/−*^ and wild-type mice. Genetic and pharmacological ablation of the IFN-I signaling pathway protected against virus-induced liver damage. These results delineate IFN-I mediated oxidative stress as a key mediator of virus-induced liver damage and describe a mechanism of innate-immunity-driven pathology, linking IFN-I signaling with antioxidant host defense and infection-associated tissue damage.

**Video Abstract:**

## Highlights

• Viral infection leads to redox dysregulation including the downregulation of SOD1
• *Sod1*^*−/−*^ mice exhibit aggravated viral hepatitis, which is rescued by antioxidants
• IFN-I signaling via STAT1 drives SOD1 downregulation and early liver damage
• Ablation of IFN-I signaling ameliorates viral hepatitis in *Sod1*^*−/−*^ and WT mice

## Graphical Abstract

**Figure:**
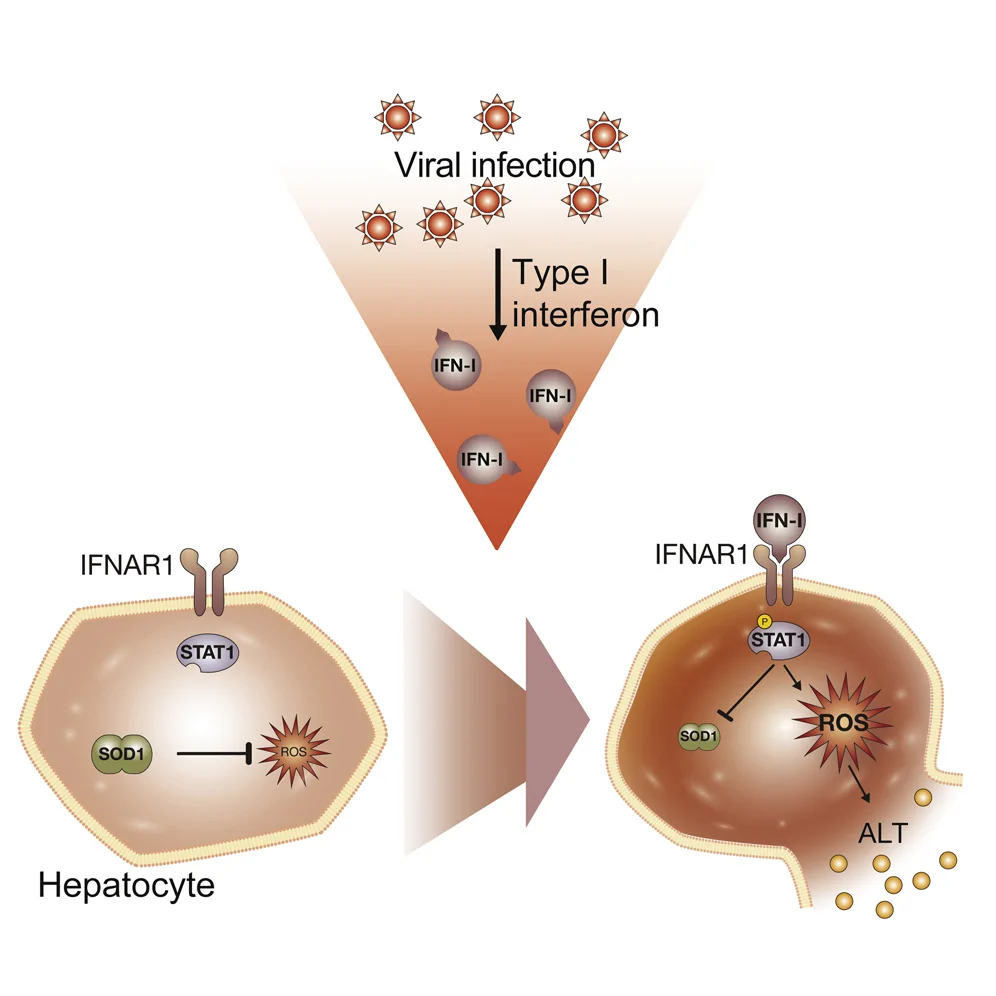

## Teaser

Bergthaler and colleagues show that superoxide dismutase 1 protects the liver from type I interferon-driven oxidative damage in viral hepatitis. Liver damage was mediated by hepatocyte-intrinsic IFNAR1-STAT1 signaling.

## Introduction

More than 500 million people worldwide are infected with Hepatitis B virus (HBV), Hepatitis C virus (HCV), or other hepatotropic viruses. These viral infections often lead to liver damage and associated complications such as advanced liver fibrosis, cirrhosis, and hepatocellular carcinoma, which cause substantial morbidity and mortality (Guidotti and Chisari, 2006, Park and Rehermann, 2014). The complex pathology of viral hepatitis is driven by multiple viral and host factors interacting with various immune cell populations and cytokines such as type I interferon-I (IFN-I) (Park and Rehermann, 2014, Schoggins et al., 2011). Together, these determinants mediate the antiviral response, but they also lead to subsequent immunopathology and tissue damage (Guidotti and Chisari, 2006, Medzhitov et al., 2012, Rouse and Sehrawat, 2010). Yet, the mechanisms involved are largely unknown.

Perturbations in several metabolic and cellular stress pathways induced by viral infections have been associated with liver disease (Drakesmith and Prentice, 2008, Koike and Moriya, 2005, Sheikh et al., 2008, Stauffer et al., 2012). Such imbalance in the host redox system resulting from infections such as HBV and HCV affects many processes governing intracellular homeostasis and signaling (Bolukbas et al., 2005, Nathan and Cunningham-Bussel, 2013, Okuda et al., 2002, Schieber and Chandel, 2014). Cells have evolved dedicated antioxidant enzymatic systems including superoxide dismutases (SODs), catalases, peroxidases, and reductases, which act as rheostats to counteract redox imbalances (Miao and St Clair, 2009, Nathan and Cunningham-Bussel, 2013, Schieber and Chandel, 2014). However, the mechanisms initiating and promoting oxidative stress and the subsequent tissue damage in viral hepatitis remain unclear.

In this study, we employed two unrelated mouse infection models to dissect host determinants of viral hepatitis, i.e., the noncytolytic lymphocytic choriomeningitis virus (LCMV) (Zinkernagel et al., 1986) and the cytolytic mouse hepatitis virus (MHV) (Cervantes-Barragan et al., 2007). We uncovered an essential role for the antioxidant SOD1 in protecting hepatocytes from virus-induced oxidative stress and cell death. Further, our data identifies IFN-I signaling as a key inducer of virus-mediated oxidative liver damage, exposing innate immunity as a driver of liver pathology. These results provide insights into the molecular pathogenesis of viral hepatitis and infection-associated tissue damage.

## Results

### Viral Infection Results in Transcriptional Regulation of Redox Pathway-Related Genes

**Figure 1:**
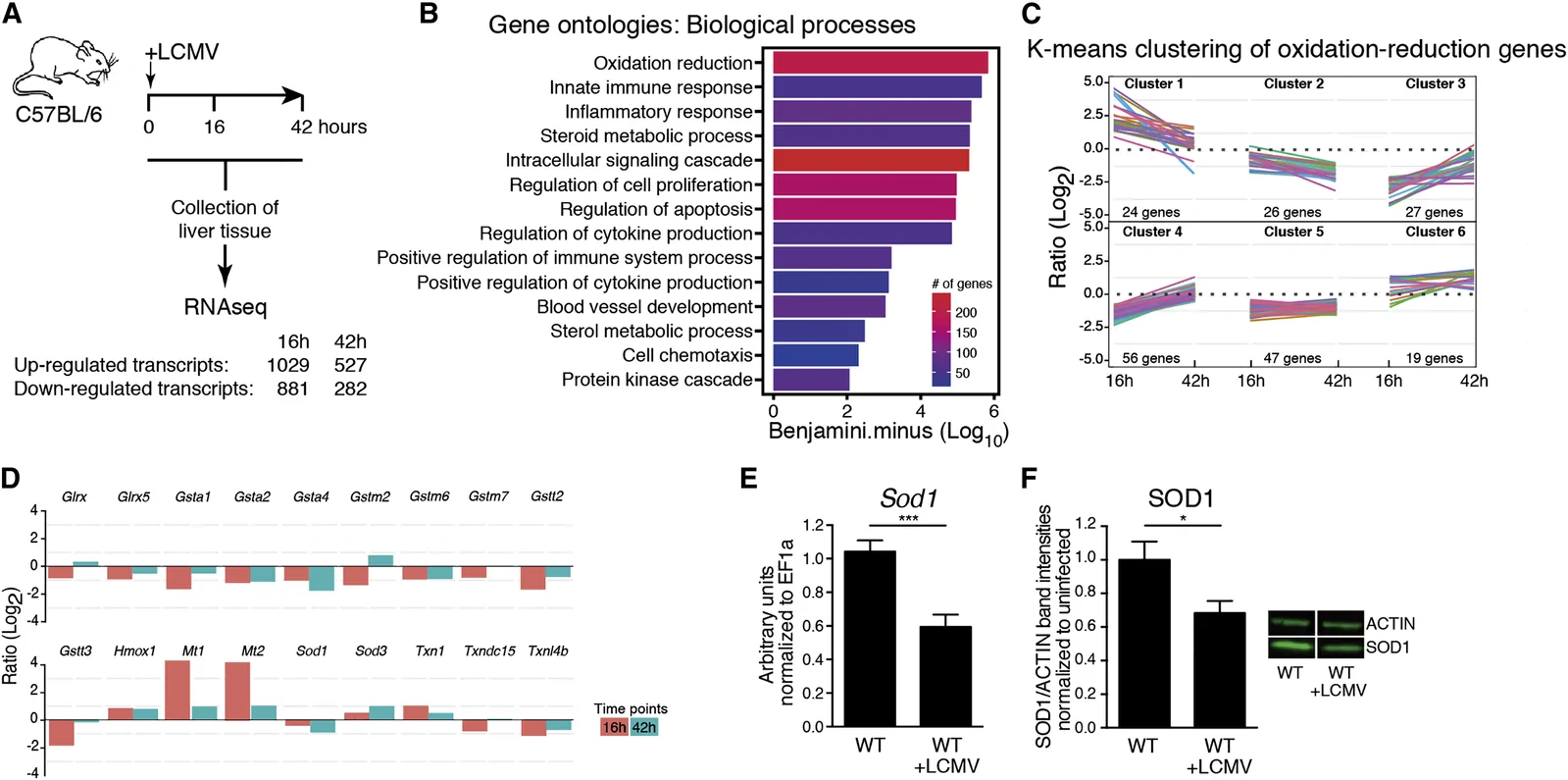
Viral Infection Results in Transcriptional Regulation of Oxidation-Reduction Pathways in the Liver (A–D) Wild-type (WT) mice were infected with LCMV. Profiling of liver tissue harvested at the indicated time points was performed by RNA-seq (n = 3 mice). (A) Workflow and summary of up- and downregulated transcripts. See also Table S1. (B) Gene ontology enrichment analysis of significantly regulated transcripts from RNaseq data. (C) K-means clustering of gene regulatory profiles from the GO term oxidation reduction. Individual genes are represented by different colored lines. See also Table S2. (D) Selection of differentially regulated genes involved in oxidation-reduction related processes (Experimental Procedures). (E) mRNA expression of *Sod1* was determined by real-time PCR from liver tissue of WT mice that were either left uninfected or infected with LCMV 44 hr previously (n = 7–12 mice per group pooled from three independent experiments). (F) Western blot for SOD1 and actin were performed from liver lysates of WT mice that were either left uninfected or infected with LCMV 44 hr previously (representative results are shown). Relative protein ratios of SOD1 to actin were quantified by LI-COR (n = 11 mice per group pooled from three independent experiments). Statistical significance was calculated by unpaired t test (E and F). Symbols represent the mean ± SEM.

To obtain an unbiased view of global gene expression in the liver in the early phase of a chronic viral infection, we infected wild-type (WT) mice with LCMV strain clone 13 and performed transcriptional profiling of liver tissue at different time points by RNA-seq (Figure 1A, Table S1). The differentially up- or downregulated transcripts were subjected to gene ontology (GO) analysis. As expected, genes involved in innate immune and inflammatory responses were significantly overrepresented (Figure 1B). The most highly enriched GO term was related to oxidation-reduction processes. Further analysis by k-means clustering revealed distinct patterns of transcriptional up- and downregulation among this group of transcripts (Figure 1C, Table S2), which included genes with antioxidant function such as glutathione S-transferases, hemoxygenase, and metallothioneins, as well as SODs (Figure 1D, Table S1). The SOD family members SOD1, SOD2, and SOD3 are crucial scavengers of O_2_^−^ (Miao and St Clair, 2009). SOD1 (also known as Cu/Zn-SOD) is ubiquitously expressed, localized in the cytoplasm, nucleus and mitochondrial intermembrane space and has been linked to human diseases such as amyotrophic lateral sclerosis (Miao and St Clair, 2009). Yet, little is known about the role of SOD enzymes in the context of infection. Decreased levels of SOD1 were found in patients chronically infected with HCV (Diamond et al., 2012, Levent et al., 2006), in HCV-induced hepatocellular carcinoma (Dillon et al., 2013, Megger et al., 2013), as well as in HBV-associated cancer tissue (Kim et al., 2003). This coincided with our observation that infection with LCMV resulted in a downregulation of SOD1 expression at the RNA (Figures 1D and 1E) and protein level (Figure 1F).

### SOD1 Deficiency Leads to Aggravated Liver Damage upon LCMV Infection

**Figure 2:**
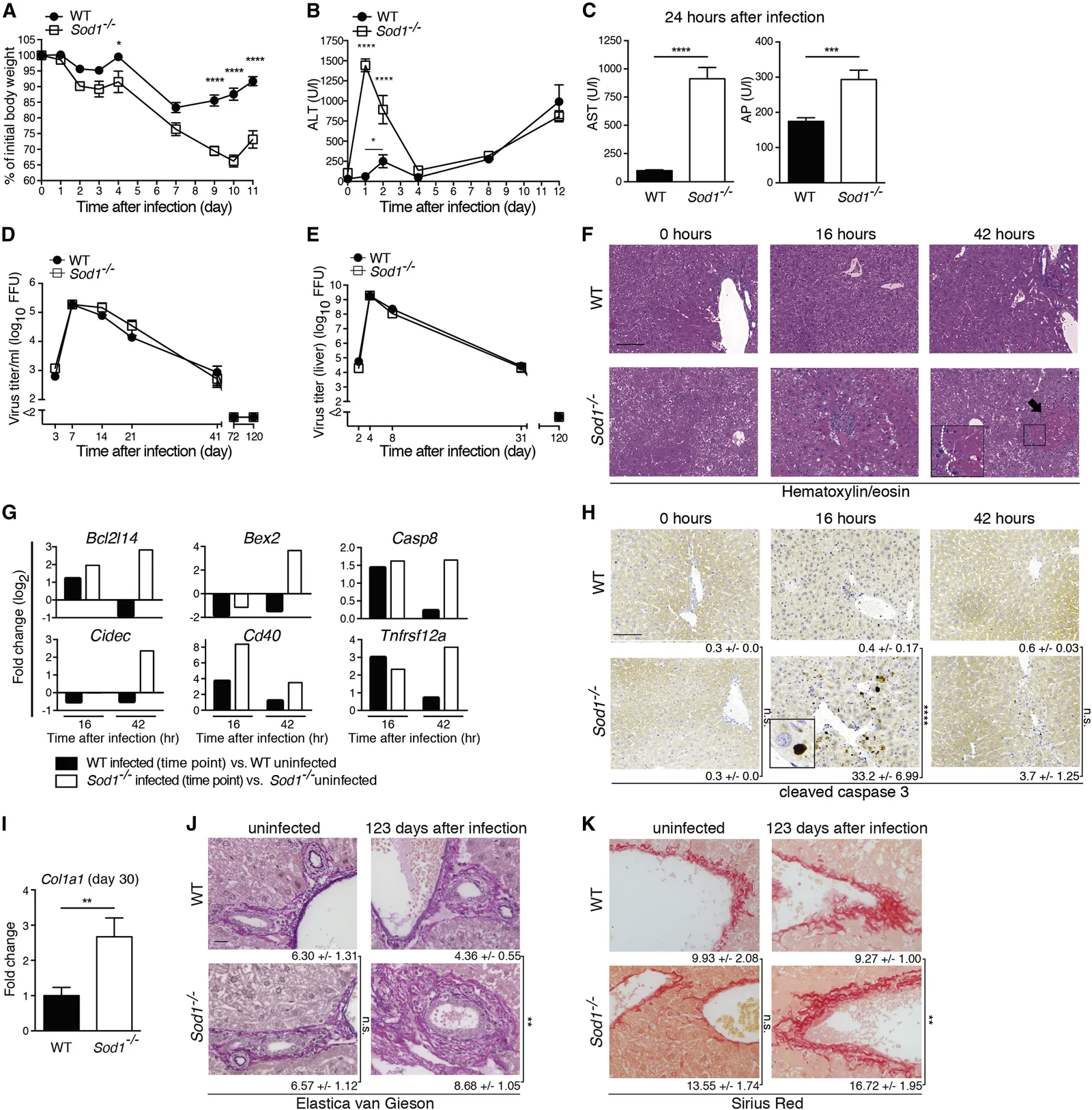
SOD1 Deficiency Leads to Aggravated Liver Damage upon LCMV Infection (A–K) WT and *Sod1*^*−/−*^ mice were infected with LCMV. (A) Body weight was monitored after LCMV infection (n = 4 mice per group). (B) Serum kinetics of alanine aminotransferase (ALT) was measured after LCMV infection (n = 10 mice per group). (C) Aspartate aminotransferase (AST) and alkaline phosphatase (AP) were analyzed 24 hr after infection (n = 10 mice per group). One out of ≥ two similar experiments is shown. (D and E) Viral loads from blood (D) and liver (E) were determined by focus-forming assay (n = 3–7). (F) Liver sections were stained for hematoxylin/eosin (H/E) (n = 3 mice per group, scale bar represents 200 μm). Representative images are shown. Pathologic lesions are highlighted by arrow and insert. (G) Comparison of a subset of RNA-seq-derived significantly differentially regulated genes involved in cell death is shown from infected versus uninfected WT or *Sod1*^*−/−*^ mice (n = 3 mice per group). (H) Liver sections were stained for cleaved caspase-3 and numbers of positive cells are shown as mean ± SEM (n = 3 mice per group, scale bar represents 200 μm). Representative images are shown. Insert shows a hepatocyte positive for cleaved caspase-3. (I) *Col1a1* mRNA was determined by real-time PCR in liver tissue 30 days after infection and fold-change was calculated between infected WT and *Sod1*^*−/−*^ mice (n = 10 mice per group, pooled from three experiments). Liver tissue was stained by Elastica van Gieson (J) and Sirius Red. (K). Pictures are representative of 9 or 10 infected mice per genotype (pooled from two independent experiments collected on day 103 and day 123, respectively, after LCMV infection) and n = 5 uninfected mice per genotype (scale bars represent 20 μm). Numbers represent the means ± SEM derived from automated quantification (% of area) based on the analysis of 5 high-power fields per sample. Statistical significance was calculated by two-way ANOVA (A, B, D, E, H) or by unpaired t test (C, I–K). Symbols/bars represent the mean ± SEM.

To investigate whether SOD enzymes contribute to viral hepatitis, we infected *Sod1*^*−/−*^, *Sod2*^*+/−*^—a commonly used model for *Sod2* deficiency (Boelsterli and Hsiao, 2008)—and *Sod3*^*−/−*^ mice with LCMV and monitored the course of disease. *Sod1*^*−/−*^ but not *Sod2*^*+/−*^ or *Sod3*^*−/−*^ mice lost more body weight compared to WT mice, which started in the early phase of infection (Figure 2A, Figure S1A). Next, we assessed serum concentrations of alanine aminotransferase (ALT), a routinely used clinical parameter of hepatitis. Again, *Sod1*^*−/−*^ mice, but neither *Sod2*^*+/−*^ nor *Sod3*^*−/−*^ mice, showed highly elevated concentrations of ALT (Figure 2B, Figure S1B) within the first 2 days of infection. We also measured two alternative parameters for hepatitis, aspartate aminotransferase (AST) and alkaline phosphatase (AP), and found elevated concentrations in *Sod1*^*−/−*^ compared to WT mice (Figure 2C). In addition, we observed an early increase of ALT in WT mice upon LCMV infection (Figure 2B). The serum concentrations of blood urea nitrogen and creatinine, which represent parameters of kidney damage, were comparable between *Sod1*^*−/−*^ and WT mice upon LCMV infection (Figure S1C), suggesting a non-generalized pathogenesis that is primarily affecting the liver. SOD2 and SOD3 were dispensable for liver protection in our experiments, which might be due to lower expression in the liver (Marklund, 1984) or different biological properties including metal cofactors and subcellular localization. To study the effects of the virus infection dose on the observed pathology, we infected *Sod1*^*−/−*^ and WT mice with either a low dose of LCMV strain clone 13 or with another LCMV strain ARM that is usually cleared within 8 days. In either case we observed increased hepatitis in *Sod1*^*−/−*^ mice compared to WT mice (Figure S1D), suggesting that the pathology is independent of the infection inoculum and LCMV strain. Together, our results indicate that SOD1 plays a non-redundant protective role in viral hepatitis and liver damage.

Upon infection *Sod1*^*−/−*^ and WT mice showed comparable viral loads in blood (Figure 2D), liver (Figure 2E), and spleen and kidney (Figure S1E), which argued against a role for SOD1 in virus control. Histological analysis of infected liver tissue revealed pathologic lesions in infected *Sod1*^*−/−*^ mice, which were absent in infected WT mice and in uninfected *Sod1*^*−/−*^ and WT mice (Figure 2F). This was associated with increased expression of cell-death-associated genes (Figure 2G) and more cleaved caspase-3 positive hepatocytes in the liver at 16 hr after infection (Figure 2H), indicating the rapid activation of apoptotic pathways in infected *Sod1*^*−/−*^ mice. Thirty days after infection with LCMV strain clone 13 *Sod1*^*−/−*^ mice showed fibrotic processes in the liver tissue as indicated by increased *Col1a1* mRNA expression (Figure 2I). A similar increase of *Col1a1* expression was observed also upon infection with the acute LCMV strain ARM (Figure S1F). More than 100 days after infection, *Sod1*^*−/−*^ mice showed recovered body weight compared to WT mice (Figure S1G) and comparable residual viral RNA in the liver (Figure S1H). Histopathological analyses of liver tissue for H/E and cleaved caspase 3 revealed no differences between *Sod1*^*−/−*^ and WT mice (Figure S1I). Yet, we found increased levels of 8-oxoguanine (8-oxoG), a marker for oxidative damage, in the liver tissue of *Sod1*^*−/−*^ mice at 123 days after infection (Figure S1I). Oxidative stress is considered to play a pathogenic role in liver fibrosis (Parola and Robino, 2001, Sánchez-Valle et al., 2012). In line with this and our *Col1a1* expression data, *Sod1*^*−/−*^ mice showed an accumulated deposition of collagen fibers compared to WT mice as detected by Elastica-van Gieson (Figure 2J) and Sirius Red staining (Figure 2K). Together, this indicated that *Sod1*^−/−^ mice exhibited increased fibrotic changes during the late phase of infection compared to WT mice.

To investigate whether the SOD1-mediated protection of the liver constituted a general host mechanism during viral infections, we infected *Sod1*^*−/−*^ and WT mice with the cytolytic murine coronavirus MHV. Similar to our previous findings with the noncytolytic LCMV, the lack of SOD1 resulted in increased body weight loss (Figure S1J) and elevated concentrations of ALT (Figure S1K). Further, we observed more histopathological lesions after infection (Figure S1L) despite similar viral loads in the liver (Figure S1M). Together, these results suggest that SOD1 plays a general protective role in the liver during viral infection.

### SOD1 Deficiency Results in Oxidative Stress-Induced Liver Damage upon Viral Infection

**Figure 3:**
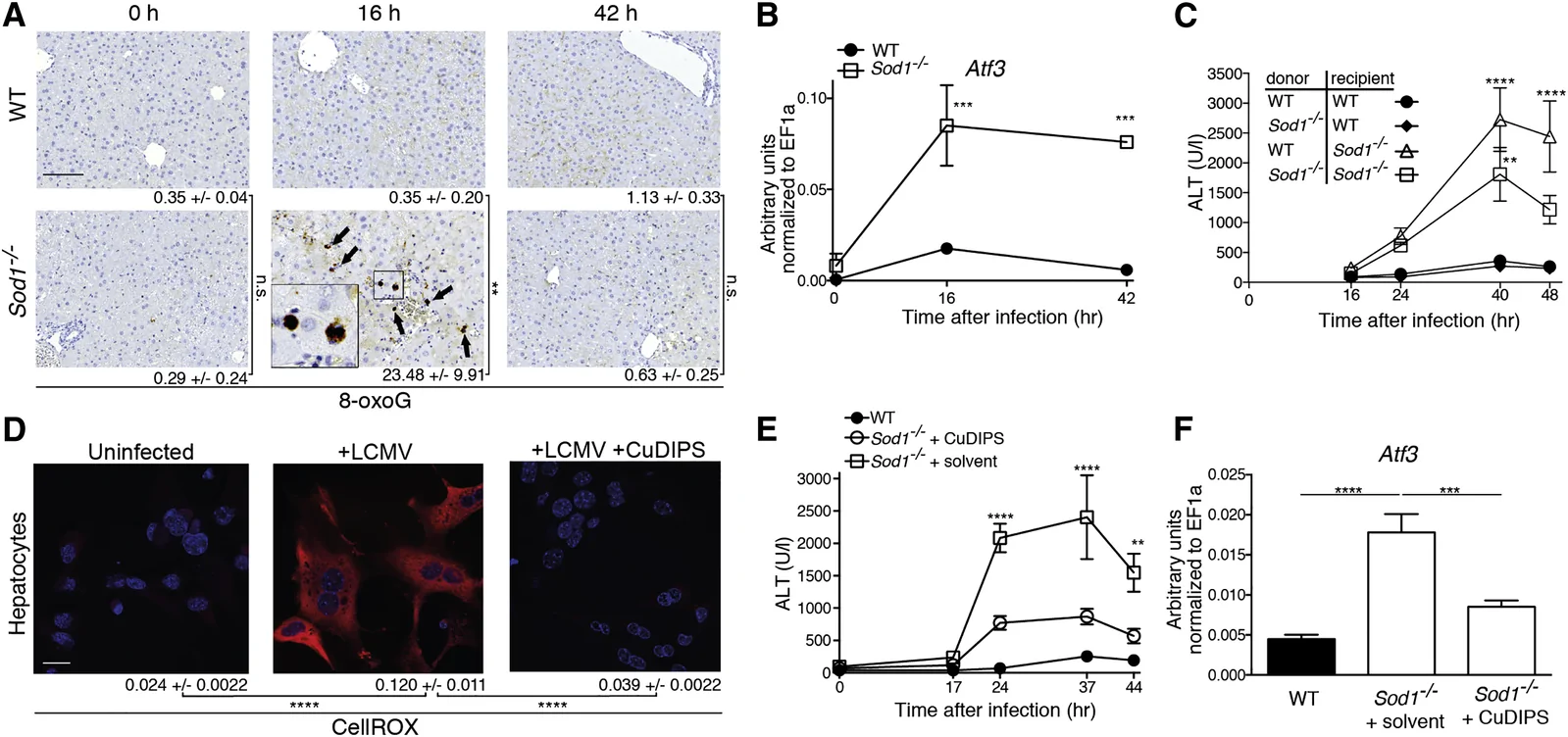
SOD1 Deficiency Leads to Oxidative Stress-Induced Liver Damage upon Viral Infection (A) Liver sections were stained for 8-oxoguanine (8-oxoG) from uninfected or LCMV infected WT and *Sod1*^*−/−*^ mice. Arrows indicate 8-oxoG positive cells. Numbers of positive nuclei are shown as mean ± SEM (n = 3 mice per group, scale bar represents 200 μm). Representative images are shown. (B) WT and *Sod1*^*−/−*^ mice were infected with LCMV. *Atf3* mRNA levels were determined in liver tissue before and after infection by real-time PCR (n = 3 mice per group). One out of ≥ two similar experiments is shown. (C) Bone marrow-chimeric mice were generated by reciprocal transfer of *Sod1*^*−/−*^ and WT genotypes. Serum ALT levels of mice infected with LCMV are shown (n = 5-8 mice per group pooled from two experiments). p values are derived from the comparison to the control group of WT→WT mice. (D) Primary hepatocytes from WT mice were left uninfected or infected with LCMV (MOI 5) +/− treatment with 10 μM CuDIPS before staining with CellROX. Scale bar represents 20 μm. Representative images are shown. Quantification was performed by CellProfiler and numbers represent mean ± SEM. One out of ≥ two similar experiments is shown. (E and F) WT and *Sod1*^*−/−*^ mice, which received either 10 mg/kg body weight CuDIPS or solvent, were infected with LCMV and (E) serum levels of ALT (n = 12 mice per group, pooled from three independent experiments) and (F) *Atf3* mRNA in liver tissue were measured 44 hr after infection (n = 8 mice per time point, pooled from two independent experiments). Statistical significance was calculated by Two-way (A–C, E) or one-way (D and F) ANOVA with Bonferroni correction. Symbols represent the mean ± SEM.

To determine whether oxidative damage was responsible for the exacerbated liver pathology observed in infected *Sod1*^*−/−*^ mice, we adopted several complementary approaches. First, we stained liver tissue of uninfected *Sod1*^*−/−*^ mice for 8-oxoG and found no differences in 8-oxoG staining compared to uninfected WT mice (Figure 3A). LCMV infection, however, led to elevated staining of 8-oxoG in hepatocytes of *Sod1*^*−/−*^ mice at 16 hr after infection (Figure 3A), confirming increased virus-induced oxidative damage in the liver. Likewise, we detected elevated mRNA expression of *Atf3* (Figure 3B), encoding a transcription factor that is induced by reactive oxygen species (ROS) and plays an important role in immunoregulation (Gilchrist et al., 2006, Hoetzenecker et al., 2012). The observed transient cellular damage as seen by histological staining for 8-oxoG and cleaved caspase 3 at 16 hr after infection might be due to refractoriness of JAK-STAT signaling after sustained IFN-I signaling in liver tissue during viral infection (Sarasin-Filipowicz et al., 2009). Thus, SOD1 is required to prevent oxidative damage in the liver upon viral infection.

We next aimed to identify the cellular compartments that require SOD1 to protect against oxidative damage. Bone-marrow-chimeric mice were generated by reciprocal transfer of *Sod1*^*−/−*^ and WT genotypes followed by administration of liposomal clodronate to deplete remaining radioresistant macrophages. Chimerism was confirmed in liver and spleen by using a congenic marker (Figures S2A and S2B). Upon LCMV infection, *Sod1*^*−/−*^→*Sod1*^*−/−*^ and WT→*Sod1*^*−/−*^, but not WT→WT nor *Sod1*^*−/−*^→WT chimeric mice exhibited elevated concentrations of ALT (Figure 3C). This result indicates an essential role for SOD1 in the non-hematopoietic compartment, of which hepatocytes comprise the major cell population in the liver.

In support of these findings, ROS production was also observed in vitro in primary mouse hepatocytes upon LCMV infection by staining with the oxidation-sensitive fluorogenic probe CellROX Deep Red Reagent (CellROX) (Figure 3D, Figures S2C and S2D), which was reversed by treatment with the antioxidant copper(II) (3,5-diisopropyl salicylate)4 (CuDIPS), a non-peptide O_2_^−^ scavenger that mimics SOD1 activity (Laurent et al., 2004). To test the potential effects of antioxidant treatment on virus-induced liver damage, we assessed the effect of CuDIPS in vivo. This ameliorated the virus-induced increase in concentrations of ALT in *Sod1*^*−/−*^ mice upon infection (Figure 3E) and led to a reduction of *Atf3* mRNA expression (Figure 3F). Together, these data reveal that oxidative stress in hepatocytes plays a fundamental role in the observed virus-induced liver pathology.

### T Cells and NK Cells Are Not Involved in the Virus-Induced SOD1-Dependent Liver Pathology

**Figure 4:**
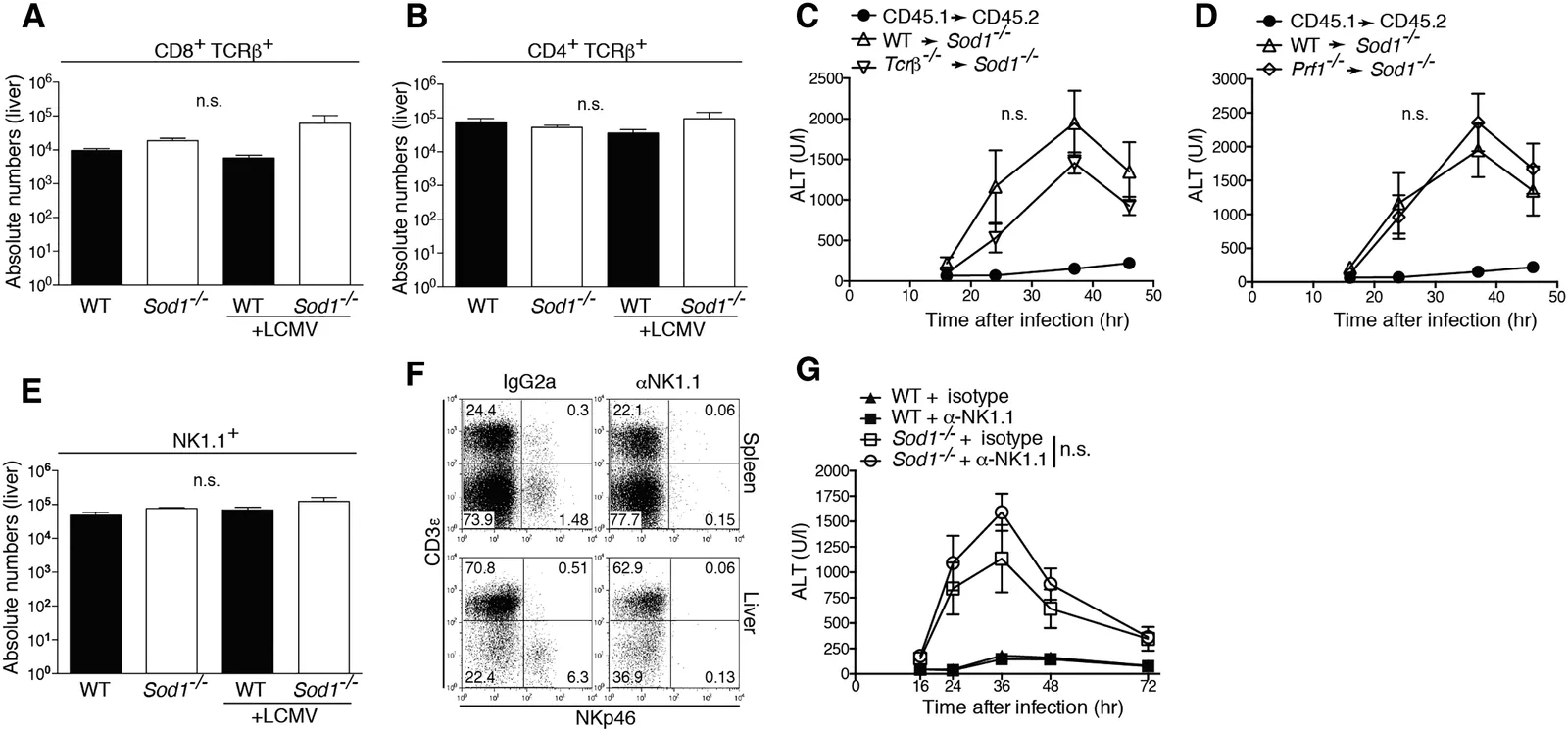
T and NK Cell Independent Liver Pathology in *Sod1*^−/−^ Mice upon Viral Infection (A and B) WT and *Sod1*^*−/−*^ mice were infected with LCMV. 24 hr after infection, (A) CD8 T cells (CD8^+^ TCRβ^+^) and (B) CD4 T cells (CD4^+^ TCRβ^+^) were enumerated in the liver (n = 4-5 mice per group). Absolute numbers are shown. (C) *Tcrb*^*−/−*^ or (D) *Prf1*^*−/−*^ (*perforin 1*) as well as WT bone marrow was transferred into irradiated *Sod1*^*−/−*^ donor mice (n = 6 mice per group from two pooled experiments). Serum levels of alanine aminotransferase (ALT) were measured upon infection with LCMV. (E) Natural killer cells (NK1.1^+^) were quantified in the liver 24 hr after LCMV infection (n = 4-5 mice per group). (F) NK cells were depleted in WT and *Sod1*^*−/−*^ mice with the anti-NK1.1 specific antibody. Depletion of NK cells was confirmed by flow cytometry in spleen and liver. (G) *Sod1*^−/−^ and WT mice, either NK1.1 depleted or treated with isotype, were infected with LCMV and serum levels of ALT were determined (n = 4 mice per group). Statistical significance was calculated by one-way (A, B, and E) ANOVA or by two-way (C, D, and G) ANOVA with Bonferroni correction. Symbols represent mean ± SEM.

T cells play a major immunopathological role in HBV and HCV infection, as well as in the hitherto-described model of LCMV hepatitis (Guidotti and Chisari, 2006, Lang et al., 2013, Park and Rehermann, 2014, Zinkernagel et al., 1986). We found comparable numbers of infiltrating CD8^+^ and CD4^+^ T cells in the liver tissue of infected *Sod1*^*−/−*^ and WT mice at 24 hr after infection (Figures 4A and 4B). Next, we assessed T cell responses in *Sod1*^*−/−*^ and WT mice after LCMV infection and found no major differences in virus-specific CD8^+^ T cells (Figures S3A–S3I) and CD4^+^ T cells (Figures S3J–S3M). In addition, T cell receptor beta chain (*Tcrb*)^*−/−*^→ *Sod1*^*−/−*^ (Figure 4C) and perforin 1 (*Prf1*)^*−/−*^→ *Sod1*^*−/−*^ bone marrow chimeric mice (Figure 4D), lacking either αβ T cells or the hematopoietically-expressed cytolytic effector protein PRF1 respectively, exhibited liver damage similar to controls upon infection. Thus, the SOD1-dependent pathology occurs independently of T cells.

NK cells are important regulators of T cell function as well as liver inflammation (Crouse et al., 2014, Rehermann, 2013, Waggoner et al., 2012, Xu et al., 2014) and we, therefore, investigated the potential role of NK cells in the observed liver pathology. Yet, we did not detect any differences in liver-infiltrating NK cells (Figure 4E) nor found any change of pathology upon the depletion of NK cells (Figures 4F and 4G). To study the potential involvement of leucocyte populations other than T cells and NK cells, we performed a cellular profiling of liver and spleen tissue and detected comparable infiltration of inflammatory monocytes, plasmacytoid dendritic cells, neutrophils, and eosinophils at 24 hr after infection (Figures S3N–S3Q). Together, these data indicate that T and NK cells are unlikely to be causally involved in the SOD1-dependent pathology and exclude a profoundly altered recruitment of inflammatory myeloid populations to the liver, arguing in favor of a hepatocyte-intrinsic defect in infected *Sod1*^*−/−*^ mice.

### Type I Interferon Drives Oxidative Damage in the Liver

**Figure 5:**
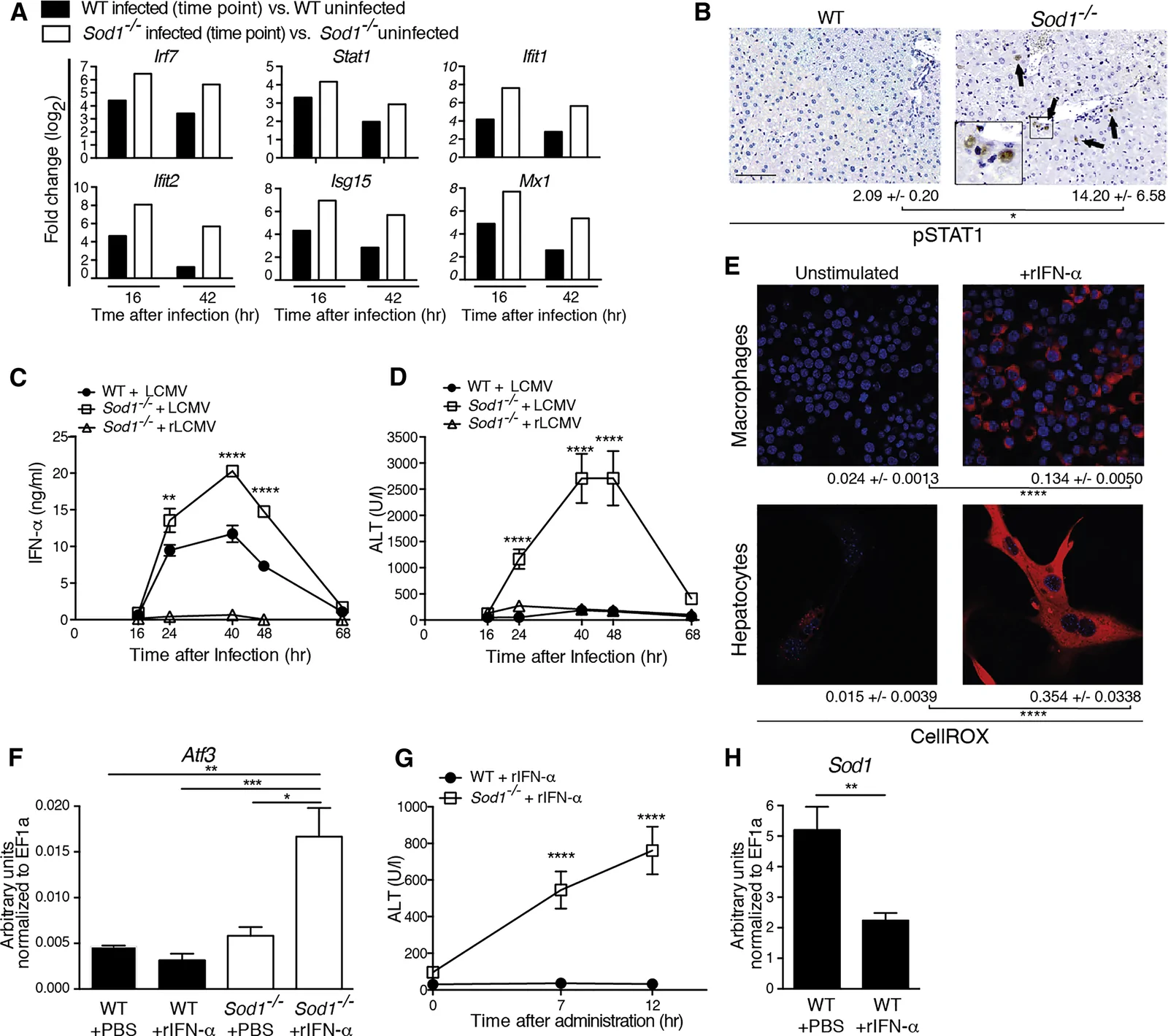
Type I Interferon Drives Oxidative Damage in the Liver (A) Comparison of a subset of RNA-seq-derived significantly differentially regulated interferon-stimulated genes from LCMV infected versus uninfected WT and *Sod1*^*−/−*^ mice (n = 3 mice per group). See also Table S1. (B) Liver sections were stained for STAT1 phosphorylation 16 hr after infection with LCMV. Arrows highlight positive nuclei. Numbers of phospho-STAT1 positive cells per mm^2^ are shown as mean ± SEM (n = 3 mice per group, scale bar represents 200 μm). Representative images are shown. (C and D) WT and *Sod1*^*−/−*^ mice were infected with LCMV and, in addition, *Sod1*^*−/−*^ mice with a rLCMV vector. (C) IFN-α and (D) ALT were measured in the serum (n = 6 mice per group). (E) RAW264.7 macrophages and primary hepatocytes from WT mice were stimulated with 2000 U/ml recombinant mouse IFN-α (rIFN-α) or left unstimulated. 24 hr later cells were stained with CellROX. Scale bar represents 20 μm. Representative images are shown. Quantification was performed by CellProfiler and numbers represent mean ± SEM. (F–H) WT respectively *Sod1*^*−/−*^ mice were treated with 100ng of rIFN-α. (F) *Atf3* and (H) *Sod1* mRNA were determined by real-time PCR in the liver 12 hr later, and (G) serum levels of ALT were measured (n = 4–6 mice per group). (C–H) one out of ≥ two similar experiments is shown. Statistical significance was calculated by two-way (B, C, D, and G) ANOVA, one-way (F) ANOVA with Bonferroni correction, or by unpaired t test (E and H). Symbols and bars represent the mean ± SEM.

In the early phase of infection, we found a more pronounced upregulation of interferon-stimulated genes in *Sod1*^*−/−*^ compared to WT mice (Figure 5A, Table S1). We also detected increased phosphorylation of signal transducer and activator of transcription 1 (STAT1), a downstream effector of IFN-I, in hepatocytes of *Sod1*^*−/−*^ compared to WT mice (Figure 5B). This prompted us to further investigate the potential role of IFN-I signaling in SOD1-dependent liver damage upon viral infection. We found that LCMV-infected *Sod1*^*−/−*^ mice exhibited higher serum concentrations of IFN-α compared to WT mice (Figure 5C), which might itself be driven by the increased phosphorylation of STAT1 upon oxidative stress (Kim and Lee, 2005). Furthermore, we infected *Sod1*^*−/−*^ mice with a replication-defective recombinant LCMV vector (rLCMV), which is capable of only a single round of infection (Flatz et al., 2010). rLCMV hardly induced any serum IFN-α (Figure 5C) and did not lead to a marked increase of ALT (Figure 5D), despite exhibiting high viral RNA loads in the liver (Figure S4A). Together, these findings demonstrate that propagating virus results in excessive oxidative damage in hepatocytes of *Sod1*^*−/−*^ mice, which was associated with the induction of IFN-I.

To address whether IFN-I induces oxidative stress, we first treated cells in vitro with rIFN-α and observed accumulation of ROS (Figure 5E). Next, we treated mice with rIFN-α, which resulted in the upregulation of the IFN-stimulated gene *Ifit1* in both *Sod1*^*−/−*^ and WT mice (Figure S4B). Importantly, rIFN-α led to increased expression of *Atf3* mRNA (Figure 5F) and was sufficient to induce hepatitis as measured by ALT in *Sod1*^*−/−*^ mice in the absence of infection (Figure 5G). In agreement with this, elevation of IFN-α induced by the neurotropic vesicular stomatitis virus (VSV) (Figure S4C) also resulted in increased concentrations of ALT in *Sod1*^*−/−*^ compared to WT mice (Figure S4D), indicating that systemic IFN-I is sufficient to induce liver damage in the absence of SOD1. Moreover, treatment with rIFN-α downregulated *Sod1* expression in WT mice (Figure 5H), similar to what we observed upon viral infection (Figures 1D–1F), suggesting a direct involvement of IFN-I signaling in the regulation of *Sod1* expression.

### Hepatocyte-Intrinsic Type I Interferon Signaling Drives Liver Damage

**Figure 6:**
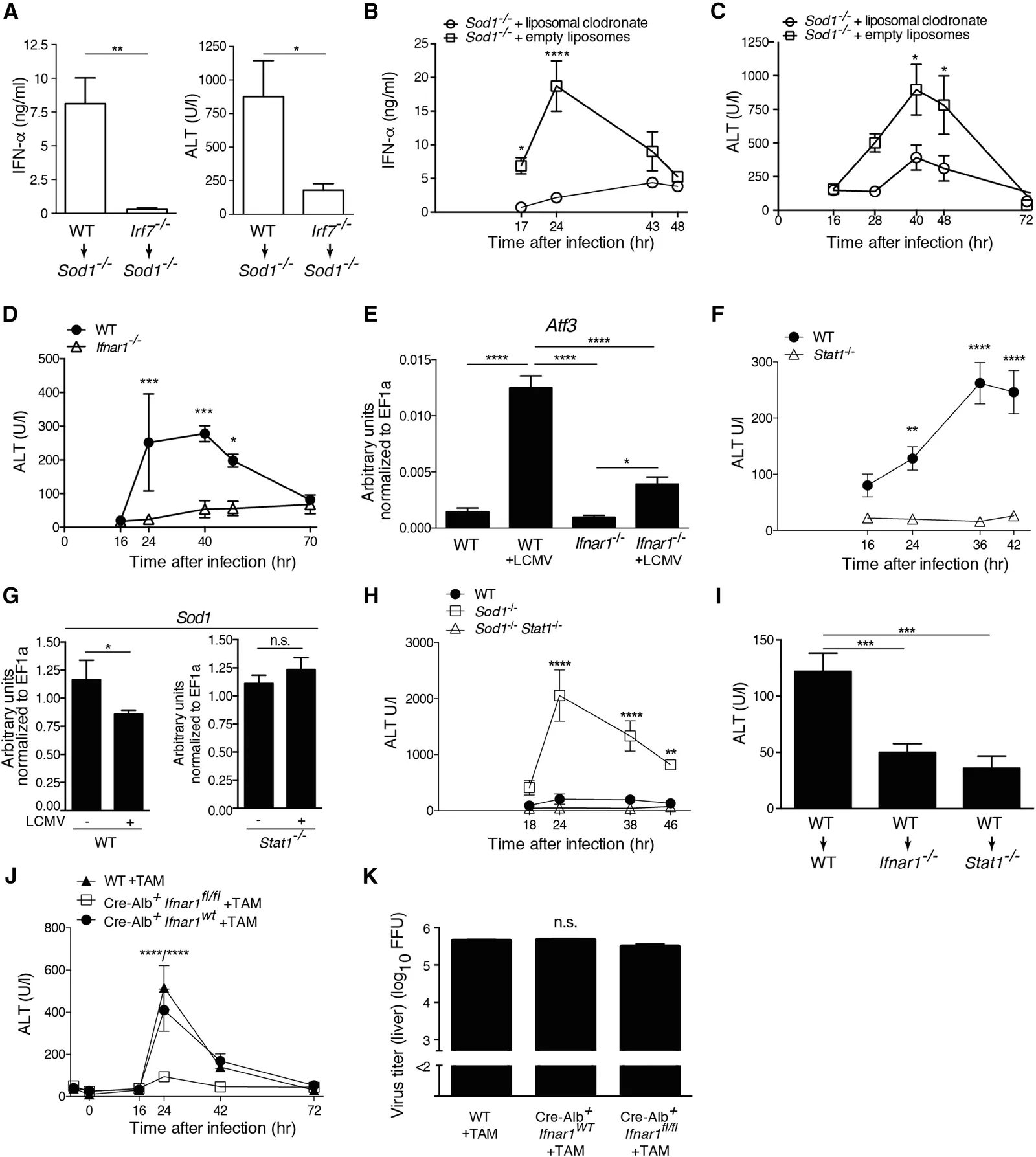
Non-hematopoietically Derived Type I Interferon Signals to IFNAR1 Expressed on Hepatocytes and Causes Liver Pathology (A) Serum levels of IFN-α (n = 6 mice) and ALT (n = 10 or 11 mice pooled from three independent experiments) of WT→*Sod1*^*−/−*^ and *Irf7*^*−/−*^→*Sod1*^*−/−*^ chimeric mice 24 hr after infection with LCMV. (B) IFN-α and (C) ALT of *Sod1*^*−/−*^ mice infected with LCMV upon treatment with liposomal clodronate or empty liposomes (n = 4 mice per group). One out of ≥ two similar experiments is shown. (D and E) WT and *Ifnar1*^−/−^ mice were infected with LCMV. Levels of (D) serum ALT and (E) *Atf3* mRNA in the liver were measured at 42 hr after infection (D and E, n = 4 mice). One out of ≥ two similar experiments is shown. (F) WT and *Stat1*^−/−^ mice were infected with LCMV and levels of serum ALT (n = 4 mice) were measured. One out of ≥ two similar experiments is shown. (G) *Sod1* mRNA (n = 4–9 mice pooled from three experiments) in the liver were measured at 42 hr after infection. (H) WT, *Sod1*^*−/−*^ and *Stat1*^−/−^*Sod1*^*−/−*^ mice were infected with LCMV and levels of serum ALT were measured (n = 4 mice per group). (I) WT→WT, WT→*Ifnar1*^*−/−*^, and WT→*Stat1*^*−/−*^ chimeric mice were infected with LCMV and levels of serum ALT at 36–39 hr after infection were measured (n = 8 mice pooled from two experiments). (J and K) WT mice, Cre-Alb ERT2 x *Ifnar1*^fl/WT^ or Cre-Alb ERT2 x *Ifnar1*^WT/WT^ (designated as Cre-Alb ERT2 × *Ifnar1*^WT^ in the graph) and Cre-Alb ERT2 x *Ifnar1*^fl/fl^ mice were administered 1 mg tamoxifen in sunflower oil i.p. each for 5 consecutive days, subsequently infected with LCMV and (J) levels of serum ALT and (K) viral loads in the liver 72 hr after infection were measured (n = 4 or 5 mice per group). Statistical significance was calculated by unpaired t test (A and G), two-way (B–D, F, H, J) or one-way (E, I, and K) ANOVA with Bonferroni correction. Symbols and bars represent the mean ± SEM.

To further dissect the role of IFN-I signaling in virus-induced oxidative liver damage, we transplanted bone marrow of WT or *Irf7*^*−/−*^ mice, which lack the master regulator of IFN-I dependent immune responses, into *Sod1*^*−/−*^ recipient mice. Upon viral infection, we observed reduced serum concentrations of IFN-α and ALT in *Irf7*^*−/−*^→*Sod1*^*−/−*^ mice compared to WT→*Sod1*^*−/−*^ mice (Figure 6A). This demonstrates that IFN-α derived from hematopoietic cells contributes to tissue damage. Previous studies have shown that a lack of phagocytes resulted in reduced concentrations of serum IFN-α (Louten et al., 2006). Indeed, *Sod1*^*−/−*^ mice treated with liposomal clodronate to deplete phagocytic cells had reduced concentrations of serum IFN-α upon infection (Figure 6B), which was accompanied by lower concentrations of ALT (Figure 6C).

To study the causative role of IFN-I in virus-induced oxidative tissue damage, we infected mice lacking IFN-α/β receptor 1 (IFNAR1) with LCMV. *Ifnar1*^−/−^ mice exhibited reduced concentrations of ALT compared to WT mice (Figure 6D) and a decreased induction of *Atf3* mRNA in the liver after infection (Figure 6E), confirming the central role of IFN-I signaling in mediating liver damage. In line with these findings, infected *Stat1*^−/−^ mice were also protected from liver damage (Figure 6F). This correlated with the absence of *Sod1* mRNA downregulation in infected *Stat1*^−/−^ mice (Figure 6G), suggesting that STAT1 signaling negatively regulates the expression of *Sod1*.

To further investigate the role of IFN-I signaling in mediating liver damage in *Sod1*^−/−^ mice, we crossed *Sod1*^−/−^ mice to *Stat1*^−/−^ mice. Indeed, these double gene-deficient mice were protected from virus-induced early hepatitis (Figure 6H). The proximity of the *Sod1* and *Ifnar1* genes (1.42cM) prevented us from generating double gene-deficient mice of this combination. Further, we also observed that WT→*Ifnar1*^*−/−*^ and WT→*Stat1*^*−/−*^ bone marrow chimeric mice were protected from early hepatitis upon LCMV infection (Figure 6I), suggesting that the liver damage is mediated by nonhematopoietic IFNAR1-STAT1 signaling. Finally, genetic ablation of *Ifnar1* specifically in hepatocytes was sufficient to confer protection (Figure 6J) despite comparable viral loads (Figure 6K). Collectively, these results provide evidence that the death of hepatocytes is mediated by cell-intrinsic IFN-I signaling through the IFNAR1-STAT1 axis.

### Blockade of Type I Interferon Signaling Ameliorates Oxidative Stress-Induced Pathology

**Figure 7:**
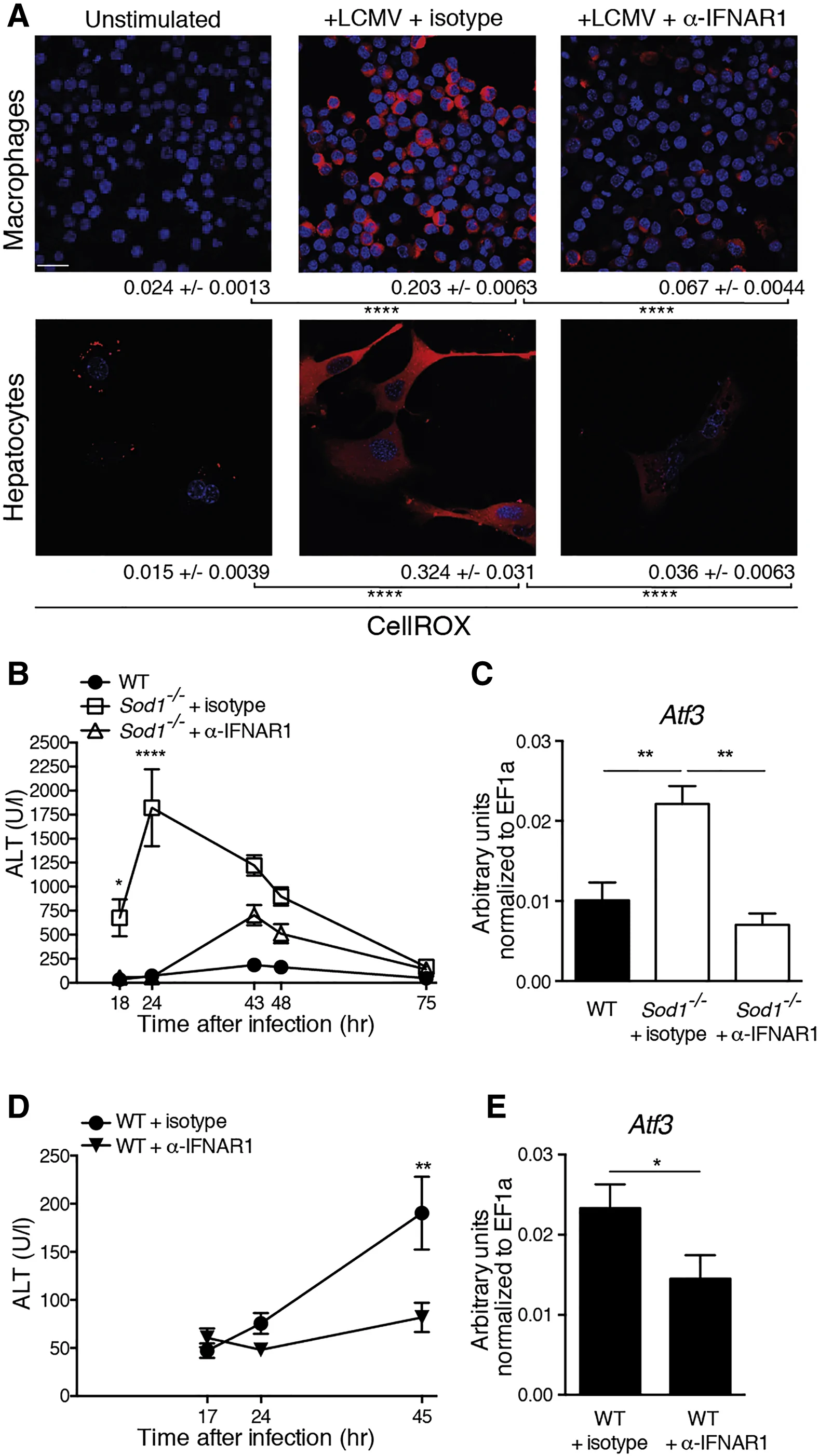
Blockade of Type I Interferon Signaling Ameliorates Oxidative Stress-Induced Liver Pathology upon Viral Infection (A) RAW264.7 macrophages and primary hepatocytes from WT mice were infected with LCMV (MOI 5) and co-treated with 20 μg/ml blocking antibody α-IFNAR1 or isotype control. 24 hr later cells were stained with CellROX. Scale bar represents 20 μm. Representative images are shown. Quantification was performed by CellProfiler and numbers represent mean ± SEM. The group of unstimulated hepatocytes is the same as used for Figure 5E. One out of ≥ two similar experiments is shown. (B) WT mice respectively *Sod1*^*−/−*^ mice, which received either isotype control or α-IFNAR1 blocking antibody, were infected with LCMV. Serum kinetics of ALT was measured (n = 4 mice per group). One out of ≥ two similar experiments is shown. (C) *Atf3* mRNA in the liver was measured 72 hr after LCMV infection from mice treated as described in (B). (D) WT mice received either isotype control or α-IFNAR1 blocking antibody and were infected with LCMV. Serum kinetics of ALT was measured (n = 9 or 10 mice pooled from two experiments). (E) *Atf3* mRNA in the liver was measured 45 hr after LCMV infection from mice treated as described in (D). Statistical significance was calculated by one-way (A and C) or by two-way ANOVA (B and D) with Bonferroni correction and (E) with unpaired t test. Symbols and bars represent the mean ± SEM.

Next, we investigated whether the pharmacological blockade of the IFN-I signaling pathway has a beneficial effect on the virus-induced oxidative tissue damage. Antibody blockade of IFNAR1 abrogated the virus-induced generation of ROS in hepatocytes and macrophages in vitro (Figure 7A), highlighting a broader relevance for IFN-I induced oxidative stress in different cell types. In line with these in vitro experiments, blockade of IFNAR1 prevented the early elevation of ALT (Figures 7B and 7D) and decreased the expression of *Atf3* mRNA in *Sod1*^*−/−*^ and WT mice upon LCMV infection (Figures 7C and 7E).

To investigate the long-term effects of transient blockade of IFNAR1, we administered the IFNAR1-specific antibody on day −1, 0, and 1 after infection as performed previously and monitored the course of chronic viral infection for 30 days. The blockade of IFNAR1 resulted in increased viremia in the early phase of infection (Figure S5A), but within 12 days the viral loads in the blood decreased to levels found in mice that had not received the blocking antibody. In line with this, comparable viral loads were found in organs 30 days after infection (Figure S5B). As shown previously in Figure 7, the blockade of IFNAR1 resulted in protection from early hepatitis, yet it subsequently led to exacerbated concentrations of ALT 12 days after infection, coinciding with improved virus control that was likely driven by T cells (Figure S5C) (Teijaro et al., 2013, Wilson et al., 2013). The transient blockade of IFNAR1 did not affect the increased expression of *Col1a1* mRNA in the liver tissue of infected *Sod1*^−/−^ mice on day 30 (Figure S5D), suggesting that early IFN-I signaling might be insufficient to drive late liver pathology. The increased *Col1a1* levels correlated with elevated *Ifit1* expression in the liver tissue of *Sod1*^−/−^ mice (Figure S5E), which might indicate a contributive role of sustained IFN-I signaling to the observed fibrotic processes. Together, these results reveal an early tissue-protective effect for the blockade of IFNAR1 and highlight the role of IFN-I in driving oxidative liver damage induced by viral infections both in *Sod1*^*−/−*^ and in WT mice.

## Discussion

These findings demonstrate that (1) SOD1 is essential in protecting hepatocytes from virus-induced damage, (2) IFN-I decreases the expression of SOD1, (3) IFN-I is necessary and sufficient to promote oxidative damage in the liver, and (4) blockade of IFN-I signaling protects from virus-induced oxidative liver damage. This functional circuit of IFN-I, SOD1, and oxidative stress provides mechanistic insights into the inflammatory and tissue-damaging processes in the liver upon viral infection, as well as a deeper understanding of the role of oxidative stress in viral hepatitis. Our data suggest a similar role for IFN-dependent processes occurring in human infections with hepatotropic and non-hepatotropic viruses, which can also result in clinically apparent liver injury (Lalazar, 2014). The observed SOD1-dependent pathology appeared to be localized predominantly to the liver, which might be due to its delicate redox status as the major organ for iron transport and storage resulting in the production of large amounts of ROS (Crichton et al., 2002).

Our study shows that the expression of SOD1 is downregulated by IFN-I signaling during viral infection and that loss of SOD1 results in oxidative damage in the liver. Together with similar observations of SOD1 downregulation in viral hepatitis in man (Diamond et al., 2012, Dillon et al., 2013, Kim et al., 2003, Levent et al., 2006, Megger et al., 2013), this implies a likely role for SOD1 in virus-driven liver pathogenesis. The molecular mechanism of how IFN-I signaling induces downregulation of *Sod1* needs further investigations and is expected to involve post-translational regulation of transcription factors such as NF-κB, AP-1, and SP1, which bind to the *Sod1* promoter (Chang and Hung, 2012, Miao and St Clair, 2009, Radaeva et al., 2002, von Marschall et al., 2003).

Liver fibrosis in chronic hepatitis C patients was found to be associated with elevated endogenous IFN-I signatures (Bièche et al., 2005, Sarasin-Filipowicz et al., 2008) and increased oxidative stress (Parola and Robino, 2001, Sánchez-Valle et al., 2012), which correlates with the data of our study. Yet, the LCMV model might not recapitulate all features of chronic liver fibrosis seen in humans, and studies with other models and/or patient samples will be required to provide further insights into the contribution of SOD1 in disease pathogenesis.

It remains to be determined whether there is a benefit to the host or whether, alternatively, the pathogen-induced oxidative stress represents simply a metabolic by-product of the IFN-I driven response. We speculate that this process might bear relevance for the metabolic rewiring of the cell, whereby the IFN-I driven transient changes in the redox status contribute to the rapidly changing bioenergetic and signaling demands as part of the antiviral state and/or of mechanisms of disease tolerance (Everts et al., 2014, Medzhitov et al., 2012, Pantel et al., 2014, Schieber and Chandel, 2014, Weinberg et al., 2015). The molecular understanding of such crosstalk between metabolic and inflammatory processes might also contribute to a better understanding of the mechanism(s) of action and side effects of IFN-I therapies in non-infectious diseases like multiple sclerosis and cancer (Reder and Feng, 2014, Sistigu et al., 2014).

Insights into this innate immunity-driven immunopathology provide a paradigm for infection-associated tissue damage by uncovering the redox system as a crucial effector downstream of the IFN-driven innate immune response. This establishes a molecular connection between cellular homeostasis, metabolism, and tissue damage in the context of viral infection and adds to the ongoing efforts to understand the pleiotropic antiviral and immunomodulatory effects of IFN-I (McNab et al., 2015, Schoggins et al., 2011, Teijaro et al., 2013, Wilson et al., 2013). Administration of targeted antioxidants and/or transient blockade of IFN-I signaling might bear liver-protective potential in the context of IFN-I responses in infectious and inflammatory diseases.

## Experimental Procedures

### Mice

*CD45.1* (Janowska-Wieczorek et al., 2001), Cre-Alb ERT2 (Schuler et al., 2004), *Irf7*^−/−^ (Honda et al., 2005), *Ifnar1*^fl/fl^ (Kamphuis et al., 2006), *Ifnar1*^−/−^ (Müller et al., 1994), *Prf1*^−/−^ (Kägi et al., 1994), *Sod1*^−/−^ (Matzuk et al., 1998), *Sod2*^*+/−*^ (Lebovitz et al., 1996), *Sod3*^*−/−*^ (Carlsson et al., 1995), *Stat1*^−/−^ (Durbin et al., 1996), *Tcrb*^*−/−*^ (Mombaerts et al., 1992) (all on a C57BL/6J genetic background), and C57BL/6J mice were bred under specific pathogen-free conditions at the Institute for Molecular Biotechnology of the Austrian Academy of Sciences in Vienna, Austria, at the Charité animal facility in Berlin, Germany, and at the animal facility of the Max Planck Institute for Infection Biology in Berlin, Germany. Experiments were performed in individually ventilated cages at the Department for Biomedical Research of the Medical University of Vienna in Vienna, Austria, at the Charité animal facility in Berlin, Germany, and at the Institute of Immunobiology, Kantonal Hospital St. Gallen in St. Gallen, Switzerland, in compliance with the respective animal experiment licenses approved by the institutional ethical committees and the institutional guidelines.

For the generation of chimeric mice, bone marrow cells were obtained from respective donor mice by flushing the femur, tibia, and fibula bones with PBS/BSA/EDTA. Recipient mice were subjected to a total irradiation of 11Gy using a Gammacell 40 B(U) (Nordion International Inc.). 1 × 10^7^ bone marrow cells from the respective donor were transferred to the recipient mice 1 day post irradiation. 4 weeks following this procedure, the chimeric mice received 200 μg of anti-CD90 antibody intraperitoneally (i.p.) to deplete any remaining peripheral T cells from the recipient. Approximately 3 weeks later, the mice received intravenously 100 μl liposomal clodronate (clodronateliposomes.com) per 10 g of bodyweight to deplete any remaining macrophages from the recipient. These mice were taken in experiment 3 weeks later to allow repopulation of macrophages. No depletions were done for the chimeric experiments shown in Figure 6I.

### Viruses

As the standard LCMV protocol, we infected mice intravenously (i.v.) with 2 × 10^6^ focus-forming units (FFU) of LCMV strain clone 13 (Bergthaler et al., 2010). In addition, we infected mice with either 2 × 10^3^ FFU (low dose) of strain clone 13 or 2 × 10^6^ FFU of strain ARM (Bergthaler et al., 2010, Bergthaler et al., 2007). For experiments with the propagation-deficient vector “rLCMV,” we infected mice i.v. with 2 × 10^5^ FFU of rLCMV/OVA (Flatz et al., 2010). Infectious titers of LCMV were determined by focus-forming assay (Bergthaler et al., 2010). For MHV experiments, mice were infected intraperitoneally with 1 × 10^3^ PFU of strain A59 (Cervantes-Barragán et al., 2009). MHV titers were determined by plaque assay (Cervantes-Barragan et al., 2007). For VSV experiments, we infected mice i.v. with 2 × 10^6^ PFU of strain Indiana. VSV titers were determined by plaque assay (Bonilla et al., 2002).

### Statistical Analysis

Results are displayed as mean ± SEM and were statistically analyzed as detailed in the figure legends using GraphPad Prism version 5 or 6. Statistically significant p values were indicated as follows: ^∗^ p ≤ 0.05, ^∗∗^ p ≤ 0.01, ^∗∗∗^ p ≤ 0.001, ^∗∗∗∗^ p ≤ 0.0001.

## Author Contributions

A. Bhattacharya and A.N.H. designed experiments, performed in vitro and in vivo studies, and wrote the manuscript. N.D., L.K., A.H., B.V., C.S., I.P., K.K., H.B., I.A., H.C.X., and P.A.L. performed in vitro and/or in vivo experiments. N.D. and L.K. contributed equally to this work. R.K.K. did bioinformatical analyses. J.C. and B.L. performed MHV experiments. D.M. and A.A.K. did histological analyses. L.F., G.S.-F., J.C., A.T., and M.L. provided reagents, analyzed data, and/or contributed to the experimental design. A. Bergthaler supervised the study, designed experiments, performed in vitro and in vivo experiments, and wrote the manuscript.

## Accession Number

The raw data from our RNaseq data are deposited at ArrayExpress (http://www.ebi.ac.uk/arrayexpress) with accession number E-MTAB-2351.

## Supplemental Information

Document S1. Figures S1–S5 and Supplemental Experimental Procedures

Document S3. Table S2

Document S4. Article plus Supplemental Information
