# Supplementary material for: Superoxide Dismutase 1 Protects Hepatocytes from Type I Interferon-Driven Oxidative Damage
Source: Immunity. Author manuscript; Available in PMC 2015 Dec 4. (PMC4658338; doi:10.1016/j.immuni.2015.10.013)
Supplement: 01 — Figure S1 (related to Figure 2) (A-B) Comparable body weight and concentration of serum ALT upon LCMV infection of Sod2+/−, Sod3−/− and WT mice. (A) body weight and (B) serum concentration of ALT were determined (n = 5 mice per group). One out of two similar experiments is shown. Statistical significance was calculated by Two-way ANOVA with Bonferroni correction. Symbols represent the mean ± S.E.M. (C) Unaltered kidney-specific blood parameter in Sod1−/− mice. WT and Sod1−/− mice were infected with LCMV and serum concentration of blood urea nitrogen and creatinine were determined (n = 3 to 9 mice, pooled from two experiments). Statistical significance was calculated by Two-way ANOVA with Bonferroni correction. Symbols represent the mean ± S.E.M. (D) Virus-induced hepatitis in Sod1−/− mice is independent of inoculum dose and virus strain. WT and Sod1−/− mice were infected with either 2×10^3^ FFU of LCMV strain clone 13 or 2×10^6^ FFU of LCMV strain ARM. Serum concentration of ALT were determined (n= 3-4 mice per group). Statistical significance was calculated by Two-way ANOVA with Bonferroni correction. Symbols and bars represent the mean ± S.E.M. (E) Comparable viral loads in Sod1−/− and WT mice. WT and Sod1−/− mice were infected with LCMV and viral loads were determined by focus-forming assay from spleen and kidney (n = 3 - 7 mice per time point pooled from four experiments). (F) Col1a1 mRNA was determined by real-time PCR in liver tissue of mice infected 2×10^6^ FFU ARM 30 days previously and fold-change was calculated between infected WT and Sod1−/− mice (n = 4-5 mice per group). Bars represent the mean ± S.E.M. (G-I) Characterization of WT and Sod1−/− mice in the late phase of infection. WT and Sod1−/− mice were infected with LCMV. (G) Body weight of mice infected 103 or 123 days previously (n = 9-10, pooled from two experiments). (H) Sod1−/− and WT mice were infected with LCMV or left uninfected, and viral RNA loads were determined in liver tissue 123 days after infe [file NIHMS66224-supplement-01.pdf]

Immunity

Supplemental Information

Superoxide Dismutase 1 Protects Hepatocytes from Type I Interferon-Driven Oxidative Damage

Anannya Bhattacharya, Ahmed N. Hegazy, Nikolaus Deigendesch, Lindsay Kosack, Jovana Cupovic, Richard K. Kandasamy, Andrea Hildebrandt, Doron Merkler, Anja A. Kühl, Bojan Vilagos, Christopher Schliehe, Isabel Panse, Kseniya Khamina, Hatoon Baazim, Isabelle Arnold, Lukas Flatz, Haifeng C. Xu, Philipp A. Lang, Alan Aderem, Akinori Takaoka, Giulio Superti-Furga, Jacques Colinge, Burkhard Ludewig, Max Löhning, and Andreas Bergthaler

Supplemental Information

Superoxide Dismutase 1 Protects Hepatocytes from Type I Interferon-Driven Oxidative Damage

Anannya Bhattacharya*, Ahmed N. Hegazy*, Nikolaus Deigendesch, Lindsay Kosack, Jovana Cupovic, Richard K. Kandasamy, Andrea Hildebrandt, Doron Merkler, Anja A. Kühl, Bojan Vilagos, Christopher Schliehe, Isabel Panse, Kseniya Khamina, Hatoon Baazim, Isabelle Arnold, Lukas Flatz, Haifeng C. Xu, Philipp A. Lang, Alan Aderem, Akinori Takaoka, Giulio Superti-Furga, Jacques Colinge, Burkhard Ludewig, Max Löhning†, Andreas Bergthaler†,‡

* Co-first authors.

† Co-senior authors.

‡ Correspondence: abergthaler@cemm.oeaw.ac.at.

Supplemental Experimental Procedures

Blood chemistry

Activities of Alanine aminotransferase (ALT), aspartate aminotransferase (AST), alkaline phosphatase (AP), blood urea nitrogen and creatinine were analyzed using a Cobas C311 Analyzer (Roche) or a 747 Automatic-Analyzer (Hitachi).

RNA isolation and real-time PCR

Total RNA was extracted from cells or homogenized tissue lysates with QIAzol lysis reagent according to the manufacturer`s instructions (Qiagen). RNA was reverse transcribed to cDNA using random primers and the First Strand cDNA Synthesis Kit (Fermentas). Real-time PCR was performed with Taqman Fast Universal PCR Mastermix (Life Tech) using the Taqman GeneExpression Assays *Atf3* (Mm00476032, Life Tech) and *Sod1* (Mm01700393, Life Tech). LCMV NP and EF1a were detected by corresponding probe and primer sets as described previously (Gilchrist et al., 2006; Pinschewer et al., 2010). Expression values are expressed as ΔCt.

RNAseq

RNA was extracted from livers of *Sod1*$^{-/-}$ and WT mice harvested 16h and 42h after infection with LCMV clone 13 (in triplicates for each time-point). For the library preparation, the TruSeq RNA sample preparation kit v2 (Illumina) was used according to the manufacturer`s protocol. Quality control analysis was performed on all samples of the cDNA library using Experion DNA Analysis chip (Biorad) and Qubit Fluorometric quantitation (Life Tech). 6 samples were pooled per lane and run on a 50bp single-end flow cell in a Hiseq2000 sequencer (Illumina).

The RNA-Seq data was processed by aligning the fastq files containing the read sequences to mouse genome (build mm9) using TopHat2. Relative fold changes were computed using Cuffdiff at an FDR of 0.05. Genes with a corrected P-value ≤ 0.05 and absolute robust Z score ≥ 1.5 were marked as differentially regulated genes. The raw data from our RNAseq data are deposited at ArrayExpress (http://www.ebi.ac.uk/arrayexpress/) with accession number E-MTAB-2351.

Bioinformatical Analysis

Enrichment for Gene Ontology – Biological Processes was carried out using DAVID analysis tool (david.abcc.ncifcrf.gov). Terms with a Benjamini-corrected p-value ≤ 0.01 were

considered as significantly enriched processes. Genes involved in oxidation-reduction were clustered into 6 distinct groups using k-means clustering method implemented using TM4 Microarray Expression Viewer (www.tm4.org). The significantly differentially regulated genes shown in **Figure 1D** were selected based on an intersection with a published list of human genes with antioxidant function (Gelain et al., 2009).

In vitro staining for oxidative stress

To detect ROS production, primary mouse hepatocytes were seeded on cover slips pre-coated with collagen and RAW264.7 macrophages were seeded on Chamber Slides Lab-Tek II (Thermo Scientific).

At the indicated time point, the cells were stained with 5µM CellROX Deep Red Reagent (Life Tech, Cat. No. C10422) for 30 min at 37 °C, washed with PBS, fixed in 4 % paraformaldehyde respectively 4 % formaldehyde for 20 min at 4 °C and counterstained with 5 µg/ml Hoechst 33258 for 7 min. Finally, the coverslips were mounted onto microscopy slides using Fluoro Mounting Medium. Quantification of immunofluorescence images with CellROX was performed based on the mean fluorescence intensity of cytoplasmic area defined by the distance from the nuclei using the CellProfiler cell image analysis software v2.0 (Carpenter et al., 2006). The size range for the pipelines was adjusted depending on the cell type.

Histology

Tissue specimens (liver) were fixed (12-48 hours at 4 °C) with 4% formaldehyde and subsequently embedded in paraffin as described previously (Bergthaler et al., 2007). Microtome sections (3 µm thick) were stained with Hematoxylin and Eosin or prepared for immunohistochemistry as follows: Endogenous peroxidases (PBS/3% H_2O_2) were neutralized and unspecific binding blocked (PBS/10% FCS). Sections were stained with primary antibodies: rabbit α-Phospho-STAT1 (detecting only phosphorylated STAT1 at position Tyrosine 701; Cell Signaling #9167), rabbit α-cleaved caspase 3 (BD Pharmingen or Cell Signalling) and mouse α-8-oxoguanine (8-oxoG) (Millipore, MAB3560) (Kitada et al., 2001) antibody. Bound primary antibodies were visualized using ready-to-use, peroxidase-based EnVision™ (Dako) secondary detection system with 3,3′-diaminobenzidine (DAB) as chromogen (haemalaun counterstaining of nuclei). Immunostained sections were registered using the slide scanner Pannoramic 250 Flash (3DHistech) at 200x magnification. Immunostained liver sections were quantified using Tissue Studio 3 software (Definiens AG, Munich, Germany). DAB positive structures were expressed as cells/nuclei per mm^2. Phospho-

STAT1 stained sections were scanned using panoramic 250 flash slide scanner (3D Histech Ltd, Hungary). Phospho-STAT1 positive nuclei in the liver were quantified using Definiens Tissue Studio® 3 software (Definiens AG, Munich, Germany) and detected nuclei were expressed as cells per mm^2.

For fibrosis stainings, 1-2 µm paraffin sections of livers were de-waxed, hydrated and stained for one hour at room temperature with Sirius Red (Polyscience, USA) and picric acid (Merck, Darmstadt). Sections were washed in acidified water (0.01N HCl), dehydrated, cleared in xylol (Roth, Karlsruhe) and mounted with corbit balsam (Hecht, Kiel). Additionally, liver sections were stained for elastic fibres with Elastica van Gieson staining kit (Merck, Darmstadt) according to manufacturer´s instructions. Nuclei appear black, elastic fibres purple and collagen red. Images were acquired using an AxioImager Z1 microscope (Carl Zeiss MicroImaging, Inc., Jena, Germany). For quantification of collagen content, five high power fields (hpf; 0.237 mm^2) per section were analysed using the module AutMess (Carl Zeiss MicroImaging, Inc.).

Histo-pathological scoring for MHV infection was performed evaluating the following criteria: i) lobular area inflammation (0 no infiltrating cells, 1 <100 infiltrating cells, 2 >100 infiltrating cells, 3 > 300 infiltrating cells), ii) lobular disarray and hepatocyte ballooning (0 nil, 1 <60%, 2 >60%) and iii) hepatocyte death (0 nil, 1 <20, 2 >20 cells).

Western blot

Protein concentration of liver lysates were determined by Coomassie Protein Assay (Thermo Scientific). Proteins were analyzed by SDS-Page using NuPAGE® Novex 4-12% Bis-Tris Gels (Life Tech), Westran® Clear signal PVDF membranes (Whatman) and the following antibodies: anti-SOD1 (Abcam #ab16831) and anti-ACTIN (Sigma #A2066). The protein size was determined with the PageRuler™ Prestained Protein Ladder (Thermo Scientific). For protein quantification, the secondary antibody donkey anti-rabbit IgG-IRDye800 (Rockland, #611-732-128) was used. The bands were quantified on an Odyssey Imager (model #9120, LI-COR) using the software Image Studio v3.1 (LI-COR).

Cytokine determination

For detection of IFNα, sera were 1:10 prediluted and analysed by ELISA using rat α-mIFN-α capture antibody (PBL Interferon Source 22100-1), rabbit α-mIFN-α detection antibody (PBL Interferon Source 32100-1), α-rabbit HRP secondary antibody (Jackson ImmunoResearch 711-036-152) and TMB solution (Life Tech 002023).

Primary mouse hepatocyte isolation

Primary hepatocytes were isolated from anesthetized mice (Ketamine/Xylazine: 1:3, 0.1 ml/10 g mouse) upon perfusion via the portal vein with washing buffer followed by digestion of the liver using liberase (Roche). Next, the liver was detached and put in a petridish containing liberase buffer. Cells were shaken out of the liver, filtered using a 70 µm cell strainer (BD Falcon) and suspended in William's E Medium GlutaMAX™ (Invitrogen) containing 10 % FCS and 1 % PSQ. The cells were allowed to settle for 20 min on ice, before the upper 25 ml containing dead cells and debris were discarded. The remaining 25 ml were spun down for 10 min at 500 rpm at 4 °C. The pellet was washed in William`s E Medium/10 % FCS/1 % PSQ and centrifuged at 500 rpm for 3 min. Finally, cells were resuspended in William`s E Medium/10 % FCS/1 % PSQ and seeded at a density of 0.75-1x10^6 cells/well on 6-well plates precoated with 50 mg/l rat tail collagen type I (BD, Cat. No. 354236) containing 8.3 % glacial acetic acid (Merck, Cat. No. 1000632500). After 3 h the medium was changed to Will E/0.5 % FCS/1 % PSQ and maintained in this medium.

Flow-cytometric analysis

Single cell suspensions of lymphoid and nonlymphoid organs were prepared by mechanical disruption. Lymphocytes were purified using Histopaque 1083 (Sigma-Aldrich) and density centrifugation (400 g at 20°C for 30 min). To prevent unspecific binding of mAb, 50 µg/ml of purified mouse and rat IgG were added to the staining mixture (Jackson Immunoresearch). Dead cells were excluded using fixable viability dye eFluor 780 (eBioscience). Subsequently, the cells were stained with the following mAbs in PBS containing 0.2% BSA and 2 mM EDTA: α-CD45 (30-F11), α-CD8a (53-6.7), α-CD11c (N418), α-F4/80 (BM8), α-MHCII (M5/114.15.2), α-NK1.1 (PK136), α-PDCA-1 (eBio927) and α-TCRβ (H57-597) were from eBioscience; α-CD45.1 (A20), α-CD45.2 (104), α-CD4 (RM4-5), α-Ly6G (1A8) and α-Ly6C (HK1.4) were from BioLegend. α-CD11b (M1/70) and α-siglec-F (E50-2440) were from BD Bioscience. Samples were acquired on a FACS LSR Fortessa or FACSCanto II (Becton Dickinson), data was analyzed with FlowJo (Tree Star).

Pharmacological treatments

Cells were treated *in vitro* with 10µM Copper (II) 3,5-diisopropylsalicylate hydrate (CuDIPS) (Sigma Aldrich, Cat. No. 341649) (Laurent et al., 2004). Mice were administered i.p. with 10mg of CuDIPS per kg body weight one day before infection, and received two additional

shots of 10mg/kg each at zero and one day after infection. Mice were administered 100ng recombinant mouse IFN-α4 i.v. (PBL Interferon Source 12115-1).

***In vivo* depletions/blockades**

To neutralize type I IFN signaling, 250μg of α-IFNAR1 antibody (clone MAR1-5A3, BioXCell) was administered i.p. one day prior to infection with two additional shots of 250μg each at zero and one day after infection. The control group was administered with a matched isotype control (clone MOPC21, BioXCell). Natural Killer (NK) cells were depleted by administration of 25 μg of α-NK1.1 (clone PK136, BioXCell) i.p. one day prior and one day after infection. The control group received mouse IgG2a isotype (clone C1.18.4, BioXCell). Phagocytic cells were depleted two days prior to infection by i.v. administration of 100μl liposomal clodronate or empty liposomes (ClodronateLiposomes.com) per 10g body weight. For tamoxifen treatment, mice were administered 1mg tamoxifen (Sigma Aldrich, Cat. No. T5648) in sunflower oil (Sigma Aldrich, Cat. No. S5007) i.p. each for 5 consecutive days.

Supplemental References

Bergthaler, A., Merkler, D., Horvath, E., Bestmann, L., and Pinschewer, D.D. (2007). Contributions of the lymphocytic choriomeningitis virus glycoprotein and polymerase to strain-specific differences in murine liver pathogenicity. The Journal of General Virology *88*, 592-603.

Carpenter, A.E., Jones, T.R., Lamprecht, M.R., Clarke, C., Kang, I.H., Friman, O., Guertin, D.A., Chang, J.H., Lindquist, R.A., Moffat, J.*, et al.* (2006). CellProfiler: image analysis software for identifying and quantifying cell phenotypes. Genome Biol *7*, R100.

Gelain, D.P., Dalmolin, R.J., Belau, V.L., Moreira, J.C., Klamt, F., and Castro, M.A. (2009). A systematic review of human antioxidant genes. Frontiers in Bioscience *14*, 4457-4463.

Gilchrist, M., Thorsson, V., Li, B., Rust, A.G., Korb, M., Roach, J.C., Kennedy, K., Hai, T., Bolouri, H., and Aderem, A. (2006). Systems biology approaches identify ATF3 as a negative regulator of Toll-like receptor 4. Nature *441*, 173-178.

Kitada, T., Seki, S., Iwai, S., Yamada, T., Sakaguchi, H., and Wakasa, K. (2001). In situ detection of oxidative DNA damage, 8-hydroxydeoxyguanosine, in chronic human liver disease. J Hepatol *35*, 613-618.

Laurent, A., Nicco, C., Tran Van Nhieu, J., Borderie, D., Chereau, C., Conti, F., Jaffray, P., Soubrane, O., Calmus, Y., Weill, B., and Batteux, F. (2004). Pivotal role of superoxide anion and beneficial effect of antioxidant molecules in murine steatohepatitis. Hepatology *39*, 1277-1285.

Pinschewer, D.D., Flatz, L., Steinborn, R., Horvath, E., Fernandez, M., Lutz, H., Suter, M., and Bergthaler, A. (2010). Innate and adaptive immune control of genetically engineered live-attenuated arenavirus vaccine prototypes. International Immunology *22*, 749-756.

Supplemental Figures

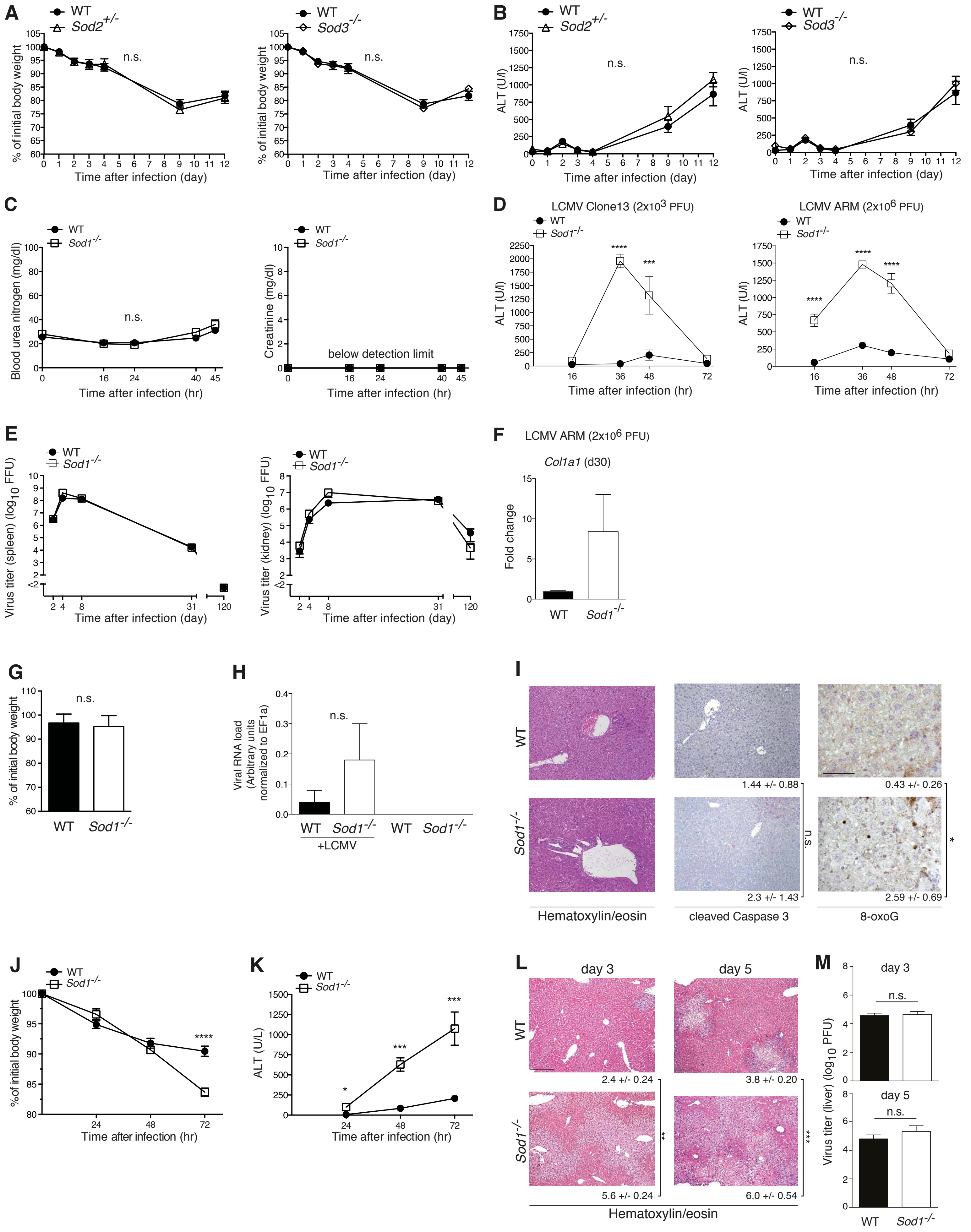
A
WT
Sod2+/-
Sod3-/-
% of initial body weight
Time after infection (day)
n.s.
B
ALT (U/l)
C
Blood urea nitrogen (mg/dl)
Creatinine (mg/dl)
below detection limit
Time after infection (hr)
D
LCMV Clone13 (2x10³ PFU)
LCMV ARM (2x10⁶ PFU)
Sod1-/-
E
Virus titer (spleen) (log10 FFU)
Virus titer (kidney) (log10 FFU)
F
Col1a1 (d30)
Fold change
G
H
Viral RNA load (Arbitrary units normalized to EF1a)
+LCMV
I
1.44 +/- 0.88
0.43 +/- 0.26
2.3 +/- 1.43
2.59 +/- 0.69
Hematoxylin/eosin
cleaved Caspase 3
8-oxoG
J
%of initial body weight
K
ALT (U/L)
L
day 3
day 5
2.4 +/- 0.24
3.8 +/- 0.20
5.6 +/- 0.24
6.0 +/- 0.54
M
Virus titer (liver) (log10 PFU)

Figure S1

Figure S1 (related to Figure 2)

(**A-B**) Comparable body weight and concentration of serum ALT upon LCMV infection of *Sod2*$^{+/-}$, *Sod3*$^{-/-}$ and WT mice. (**A**) body weight and (**B**) serum concentration of ALT were determined (n = 5 mice per group). One out of two similar experiments is shown. Statistical significance was calculated by Two-way ANOVA with Bonferroni correction. Symbols represent the mean ± S.E.M.

(**C**) Unaltered kidney-specific blood parameter in *Sod1*$^{-/-}$ mice. WT and *Sod1*$^{-/-}$ mice were infected with LCMV and serum concentration of blood urea nitrogen and creatinine were determined (n = 3 to 9 mice, pooled from two experiments). Statistical significance was calculated by Two-way ANOVA with Bonferroni correction. Symbols represent the mean ± S.E.M.

(**D**) Virus-induced hepatitis in *Sod1*$^{-/-}$ mice is independent of inoculum dose and virus strain. WT and *Sod1*$^{-/-}$ mice were infected with either 2x10^{3} FFU of LCMV strain clone 13 or 2x10^{6} FFU of LCMV strain ARM. Serum concentration of ALT were determined (n= 3-4 mice per group). Statistical significance was calculated by Two-way ANOVA with Bonferroni correction. Symbols and bars represent the mean ± S.E.M.

(E) Comparable viral loads in *Sod1*$^{-/-}$ and WT mice. WT and *Sod1*$^{-/-}$ mice were infected with LCMV and viral loads were determined by focus-forming assay from spleen and kidney (n = 3 - 7 mice per time point pooled from four experiments).

(**F**) *Col1a1* mRNA was determined by real-time PCR in liver tissue of mice infected 2x10^{6} FFU ARM 30 days previously and fold-change was calculated between infected WT and *Sod1*$^{-/-}$ mice (n = 4-5 mice per group). Bars represent the mean ± S.E.M.

(G-I) Characterization of WT and *Sod1*$^{-/-}$ mice in the late phase of infection. WT and *Sod1*$^{-/-}$ mice were infected with LCMV. (**G**) Body weight of mice infected 103 or 123 days previously (n = 9-10, pooled from two experiments). (**H**) *Sod1*$^{-/-}$ and WT mice were infected with LCMV or left uninfected, and viral RNA loads were determined in liver tissue 123 days after infection by real-time PCR (n= 3-7 mice per group). (**I**) Histological analysis of liver tissue sections of mice infected 103 or 123 days previously: H/E staining, Staining for cleaved caspase 3 positive hepatocytes (n = 9-10, pooled from two experiments), 8-oxoG staining (n = 5-7 mice per group, scale bar = 50μM). Positive cells were counted in 10 high power fields. Statistical significance was calculated by unpaired t-test (**G**, **I**) or One-way ANOVA with Bonferroni correction (**H**). Bars and values represent the mean ± S.E.M.

(J-M) SOD1 deficiency leads to aggravated liver damage upon MHV infection. WT and *Sod1*$^{-/-}$ mice were infected with MHV and (**J**) body weight and (**K**) serum concentration of ALT were

determined (n = 9 - 10 mice per group of two pooled experiments). (**L**) Liver sections 3 and 5 days after infection were stained for hematoxylin/eosin (n = 5 mice per group, scale bar = 200μm). Representative images are shown. Histopathological scores for liver damage were determined as described in the Supplemental Information. (**M**) Viral loads in the liver for 3 and 5 days after infection were determined by plaque assay (n = 5 mice per group). Statistical significance was calculated by Two-way ANOVA with Bonferroni correction (**J-K**) or by unpaired t-test (**L-M**). Symbols and bars represent the mean ± S.E.M.

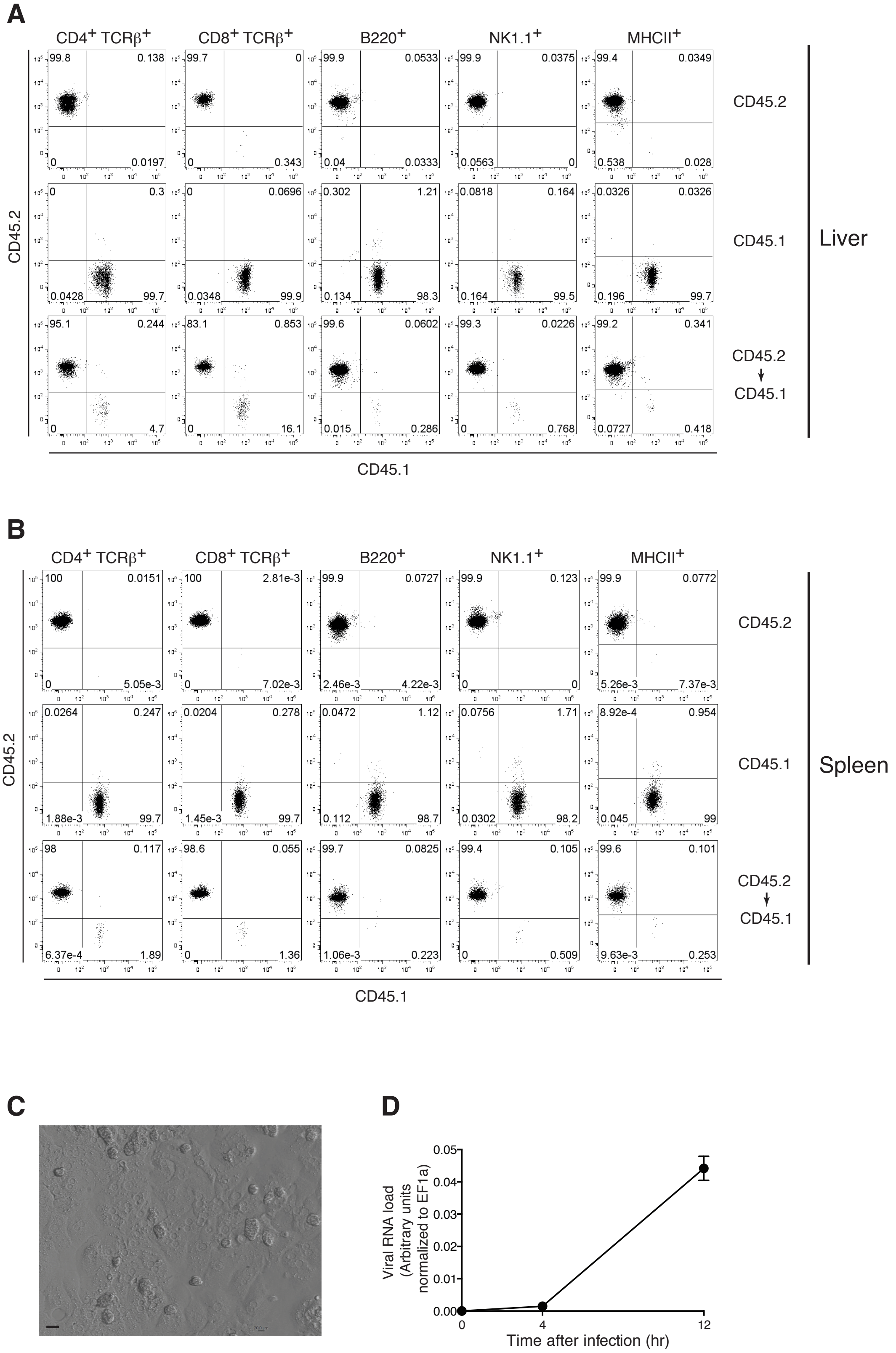
A
CD4+ TCRβ+
CD8+ TCRβ+
B220+
NK1.1+
MHCII+
CD45.2
CD45.1
CD45.2 → CD45.1
Liver
CD45.2
CD45.1
99.8 0.138 0 0.0197
99.7 0 0 0.343
99.9 0.0533 0.04 0.0333
99.9 0.0375 0.0563 0
99.4 0.0349 0.538 0.028
0 0.3 0.0428 99.7
0 0.0696 0.0348 99.9
0.302 1.21 0.134 98.3
0.0818 0.164 0.164 99.5
0.0326 0.0326 0.196 99.7
95.1 0.244 0 4.7
83.1 0.853 0 16.1
99.6 0.0602 0.015 0.286
99.3 0.0226 0 0.768
99.2 0.341 0.0727 0.418
B
CD4+ TCRβ+
CD8+ TCRβ+
B220+
NK1.1+
MHCII+
CD45.2
CD45.1
CD45.2 → CD45.1
Spleen
CD45.2
CD45.1
100 0.0151 0 5.05e-3
100 2.81e-3 0 7.02e-3
99.9 0.0727 2.46e-3 4.22e-3
99.9 0.123 0 0
99.9 0.0772 5.26e-3 7.37e-3
0.0264 0.247 1.88e-3 99.7
0.0204 0.278 1.45e-3 99.7
0.0472 1.12 0.112 98.7
0.0756 1.71 0.0302 98.2
8.92e-4 0.954 0.045 99
98 0.117 6.37e-4 1.89
98.6 0.055 0 1.36
99.7 0.0825 1.06e-3 0.223
99.4 0.105 0 0.509
99.6 0.101 9.63e-3 0.253
C
D
Viral RNA load (Arbitrary units normalized to EF1a)
0.00
0.01
0.02
0.03
0.04
0.05
0
4
12
Time after infection (hr)

Figure S2

Figure S2 (related to Figure 3)

(A-B) Successful chimerism in liver and spleen tissue. CD45.2 bone marrow was transferred into congenic CD45.1 recipient mice. Chimerism in (**A**) liver and (**B**) spleen was determined for CD4 T cells ($CD4^{+}$ $TCR\beta^{+}$), CD8 T cells ($CD8^{+}$ $TCR\beta^{+}$), B cells ($B220^{+}$), NK cells ($NK1.1^{+}$) and antigen-presenting cells ($MHCII^{+}$) by using the surface markers CD45.1 and CD45.2. Graphs are representative of three CD45.2→CD45.1 chimeric mice.

(**C-D**) Primary mouse hepatocytes are permissive to LCMV infection. (**C**) Phase contrast picture of primary mouse hepatocytes at day one of culture (scale bar = 40μm). (**D**) Primary mouse hepatocytes were infected with LCMV at a MOI of 0.02 and intracellular viral RNA loads were determined by real-time PCR (n = 3 replicate wells per time point). Symbols represent the mean ± S.E.M.

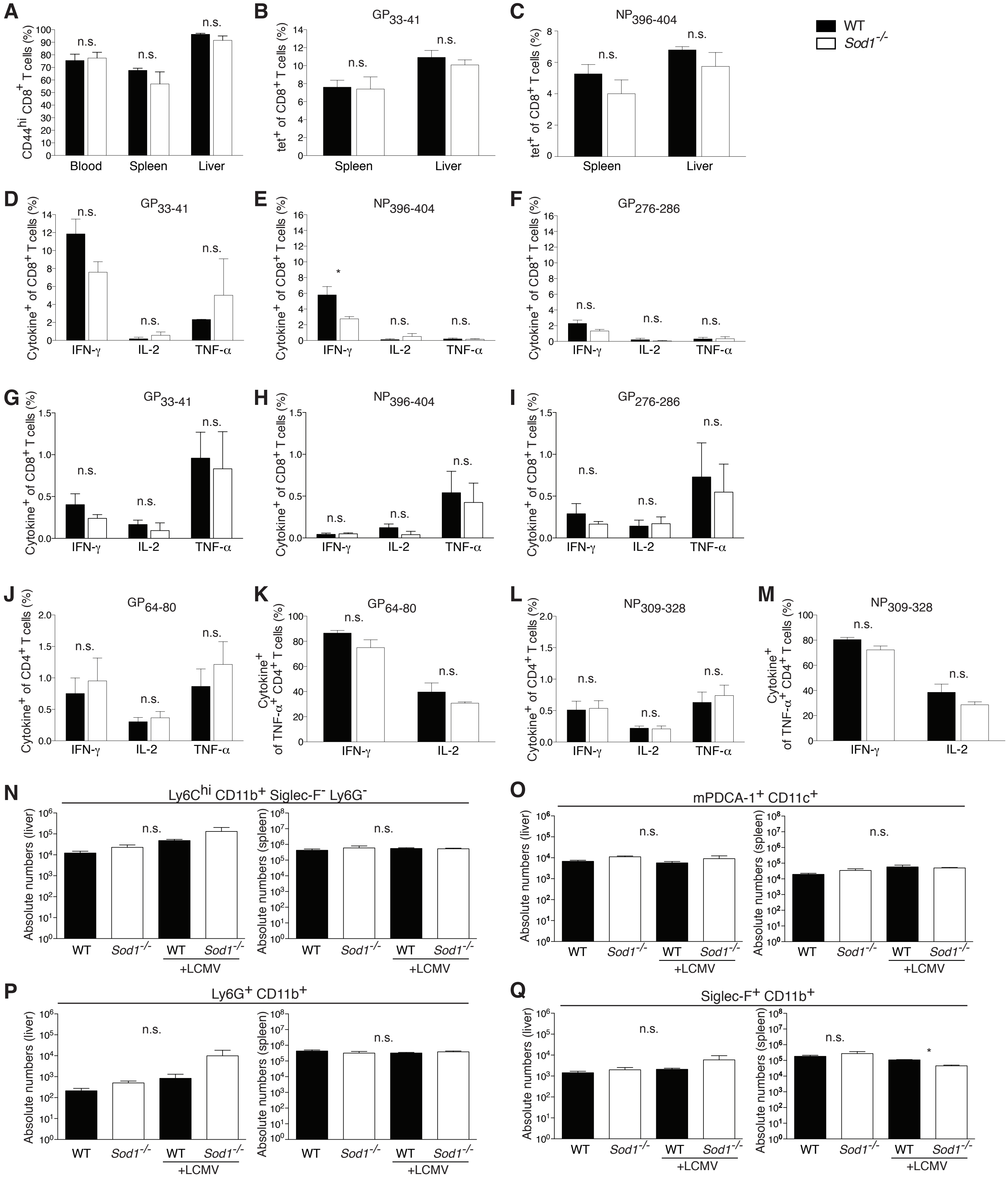

A
B
C
D
E
F
G
H
I
J
K
L
M
N
O
P
Q
WT
Sod1-/-
GP33-41
NP396-404
GP276-286
GP64-80
NP309-328
n.s.
*
CD44hi CD8+ T cells (%)
tet+ of CD8+ T cells (%)
Cytokine+ of CD8+ T cells (%)
Cytokine+ of CD4+ T cells (%)
Cytokine+ of TNF-α+ CD4+ T cells (%)
Blood
Spleen
Liver
IFN-γ
IL-2
TNF-α
Ly6Chi CD11b+ Siglec-F- Ly6G-
mPDCA-1+ CD11c+
Ly6G+ CD11b+
Siglec-F+ CD11b+
Absolute numbers (liver)
Absolute numbers (spleen)
+LCMV

Figure S3

Figure S3 (related to Figure 4)

(A-M) WT and *Sod1*$^{-/-}$ mice develop comparable CD8 and CD4 T cell responses. WT and *Sod1*$^{-/-}$ mice were infected with LCMV and CD8 T cell **(A-F; G-I)** and CD4 T cell **(J-M)** responses were enumerated by flow cytometry 8 days (**A-F**, **J-M**; n = 3 mice per group) and 31 days (**G-I**; n = 5-6 mice per group), respectively, after infection. (**A**) Total CD44^{hi} CD8 T cells and virus-specific CD8 T cell responses were measured in the indicated organs by MHC class I tetramers for the viral epitopes (**B**) GP_{33-41} and (**C**) $NP_{396-404}$. Intracellular cytokine staining of splenic CD8 T cells for IFN-γ, IL-2 and TNF-α was done following peptide stimulation *in vitro* for the viral epitopes GP_{33-41} (**D**, **G**), $NP_{396-404}$ (**E**, **H**) and $GP_{276-286}$ (**F**, **I**). Values of medium control were subtracted. Intracellular cytokine staining of splenic CD4 T cells was done following peptide stimulation *in vitro* for the viral epitopes GP_{64-80} (**J**, **K**) and $NP_{309-328}$ (**L**, **M**). Statistical significance was calculated by unpaired t-test. Bars represent the mean ± S.E.M.

(N-Q) Similar leucocyte infiltration in liver of WT and *Sod1*$^{-/-}$ mice upon LCMV infection. WT and *Sod1*$^{-/-}$ mice were infected with LCMV. 24 hours after infection the following CD45^{+} leucocyte populations were enumerated: (**N**) monocytes (Ly6C^{hi} CD11b^{+} Siglec-F^{-} Ly6G^{-}), (**O**) plasmacytoid dendritic cells (mPDCA1^{+} CD11c^{+}), (**P**) neutrophils (Ly6G^{+} CD11b^{+}) and (**Q**) eosinophils (Siglec-F^{+} CD11b^{+}). Absolute cell numbers are shown (n = 4-5 mice per group). Statistical significance was calculated by One-way ANOVA with Bonferroni correction. Bars represent the mean ± S.E.M.

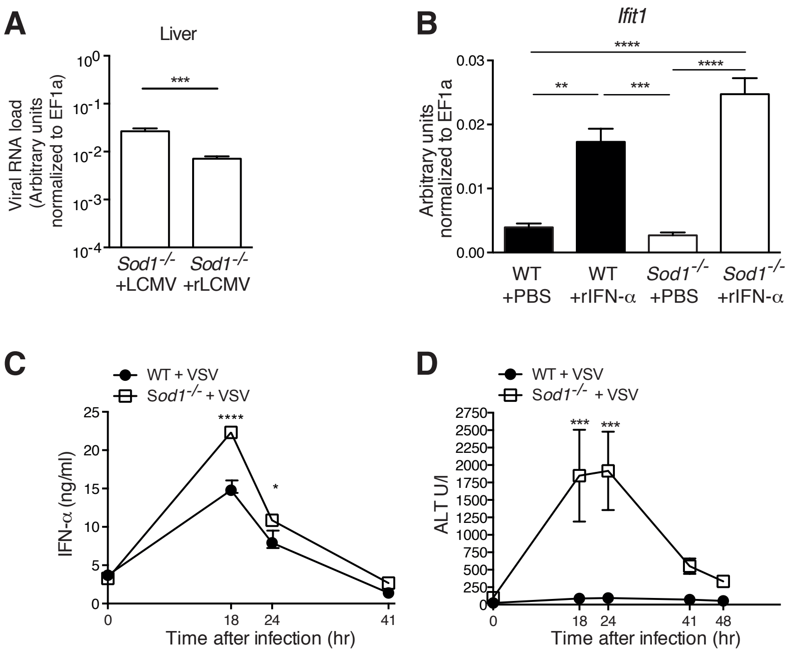
A
Liver
Viral RNA load (Arbitrary units normalized to EF1a)
***
Sod1-/- +LCMV
Sod1-/- +rLCMV
B
Ifit1
Arbitrary units normalized to EF1a
****
**
***
****
WT +PBS
WT +rIFN-α
Sod1-/- +PBS
Sod1-/- +rIFN-α
C
WT + VSV
Sod1-/- + VSV
IFN-α (ng/ml)
****
*
Time after infection (hr)
D
WT + VSV
Sod1-/- + VSV
ALT U/l
***
***
Time after infection (hr)

Figure S4

Figure S4 (related to Figure 5)

(A) Viral RNA loads in liver tissue of rLCMV-infected *Sod1*$^{-/-}$ mice. *Sod1*$^{-/-}$ mice were infected with LCMV or rLCMV, and viral RNA loads were determined in liver 72 hours after infection by real-time PCR (n= 6 mice per group). The results are representative of two similar experiments. Statistical significance was calculated by unpaired t-test. Bars represent the mean ± S.E.M.

(B) Treatment with rIFNα induces expression of *Ifit1* in the liver. WT and *Sod1*$^{-/-}$ mice were administered with 100ng of rIFNα, and levels of *Ifit1* mRNA in the liver were determined 12 hours after administration by real-time PCR (n = 4-6 mice per group). Statistical significance was calculated by One-way ANOVA with Bonferroni correction. The results are representative of two similar experiments. Bars represent the mean ± S.E.M.

(C-D) SOD1 deficiency leads to aggravated liver damage upon infection with vesicular stomatitis virus (VSV). WT and *Sod1*$^{-/-}$ mice were infected with VSV and serum concentrations of (**C**) IFNα and (**D**) ALT were determined (n = 6 mice per group). Statistical significance was calculated by Two-way ANOVA with Bonferroni correction. The results are representative of two similar experiments. Symbols represent the mean ± S.E.M.

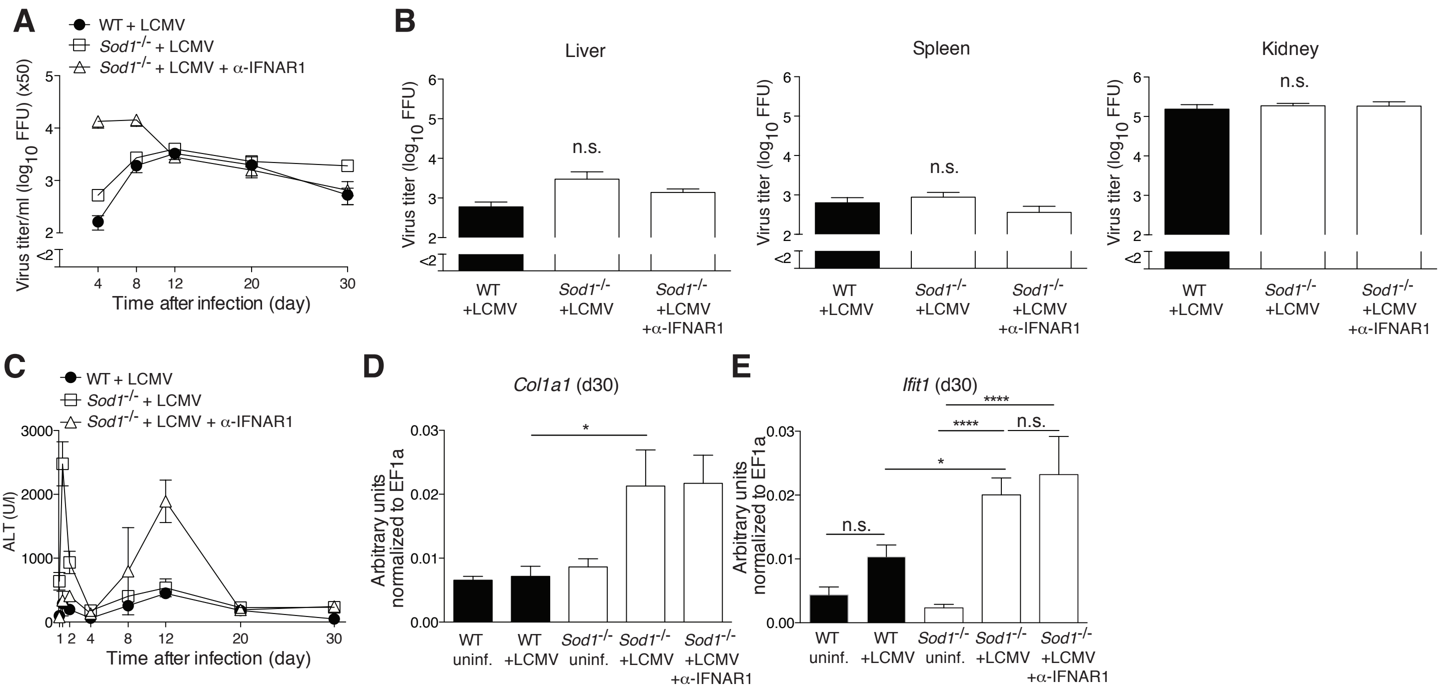
A
WT + LCMV
Sod1-/- + LCMV
Sod1-/- + LCMV + α-IFNAR1
Virus titer/ml (log10 FFU) (x50)
Time after infection (day)
B
Liver
Spleen
Kidney
Virus titer (log10 FFU)
n.s.
WT +LCMV
Sod1-/- +LCMV
Sod1-/- +LCMV +α-IFNAR1
C
ALT (U/l)
Time after infection (day)
D
Col1a1 (d30)
Arbitrary units normalized to EF1a
WT uninf.
WT +LCMV
Sod1-/- uninf.
Sod1-/- +LCMV
Sod1-/- +LCMV +α-IFNAR1
E
Ifit1 (d30)
****
n.s.
*

Figure S5

Figure S5 (related to Figure 7)

(A-E) Long-term impact of transient IFNAR1 blockade in *Sod1*$^{-/-}$ mice upon viral infection. *Sod1*$^{-/-}$ mice either with or without administration of α-IFNAR1 antibody (250 μg i.p. each on day -1, 0 and 1) and WT mice were infected with LCMV. (**A, B**) Viral loads were measured by focus forming assay in (**A**) blood and (**B**) in liver, spleen and kidney 30 days after infection. (**C**) Serum concentrations of ALT. (**D)** *Col1a1* mRNA and **(E)** *Ifit1* mRNA were determined by real-time PCR in liver tissue 30 days after infection. N = 3-8 mice per group. Statistical significance was calculated by (**A, C**) Two-way or (**B, D, E**) One-way ANOVA with Bonferroni correction. Symbols represent the mean ± S.E.M.

Supplemental Tables

Table S1 (related to Figures 1 and 5): RNAseq data from liver tissue of LCMV-infected *Sod1*$^{-/-}$ and WT mice.

Transcriptional analysis of liver tissue of *Sod1*$^{-/-}$ and WT mice 0, 16 or 42 hours after infection with LCMV (n = 3 mice per group).

Table S2 (related to Figure 1): K-means clustering of oxidation-reduction genes.

List of K-means clustering of oxidation-reduction related genes derived from RNAseq of liver tissue of *Sod1*$^{-/-}$ and WT mice (**Table S1**) after infection with LCMV.
